# Atrogin-1 promotes muscle homeostasis by regulating levels of endoplasmic reticulum chaperone BiP

**DOI:** 10.1172/jci.insight.167578

**Published:** 2024-03-26

**Authors:** Avnika A. Ruparelia, Margo Montandon, Jo Merriner, Cheng Huang, Siew Fen Lisa Wong, Carmen Sonntag, Justin P. Hardee, Gordon S. Lynch, Lee B. Miles, Ashley Siegel, Thomas E. Hall, Ralf B. Schittenhelm, Peter D. Currie

**Affiliations:** 1 Australian Regenerative Medicine Institute, Monash University, Clayton, Victoria, Australia.; 2 Department of Anatomy and Physiology, School of Biomedical Sciences, Faculty of Medicine Dentistry and Health Sciences, and; 3 Centre for Muscle Research, Department of Anatomy and Physiology, University of Melbourne, Melbourne, Victoria, Australia.; 4 Monash Proteomics and Metabolomics Facility, Monash Biomedicine Discovery Institute, Monash University, Clayton, Victoria, Australia.; 5 Institute for Molecular Bioscience, University of Queensland, Brisbane, Queensland, Australia.; 6 EMBL Australia, Victorian Node, Monash University, Clayton, Victoria, Australia.

**Keywords:** Muscle biology, Genetic diseases, Muscle, Ubiquitin-proteosome system

## Abstract

Skeletal muscle wasting results from numerous pathological conditions affecting both the musculoskeletal and nervous systems. A unifying feature of these pathologies is the upregulation of members of the E3 ubiquitin ligase family, resulting in increased proteolytic degradation of target proteins. Despite the critical role of E3 ubiquitin ligases in regulating muscle mass, the specific proteins they target for degradation and the mechanisms by which they regulate skeletal muscle homeostasis remain ill-defined. Here, using zebrafish loss-of-function models combined with in vivo cell biology and proteomic approaches, we reveal a role of atrogin-1 in regulating the levels of the endoplasmic reticulum chaperone BiP. Loss of atrogin-1 resulted in an accumulation of BiP, leading to impaired mitochondrial dynamics and a subsequent loss in muscle fiber integrity. We further implicated a disruption in atrogin-1–mediated BiP regulation in the pathogenesis of Duchenne muscular dystrophy. We revealed that BiP was not only upregulated in Duchenne muscular dystrophy, but its inhibition using pharmacological strategies, or by upregulating atrogin-1, significantly ameliorated pathology in a zebrafish model of Duchenne muscular dystrophy. Collectively, our data implicate atrogin-1 and BiP in the pathogenesis of Duchenne muscular dystrophy and highlight atrogin-1’s essential role in maintaining muscle homeostasis.

## Abstract 2

Zebrafish loss-of-function models combined with in vivo cell biology and proteomic approaches identified endoplasmic reticulum chaperone BiP as a target of the E3 ubiquitin ligase atrogin-1.

## Graphical abstract

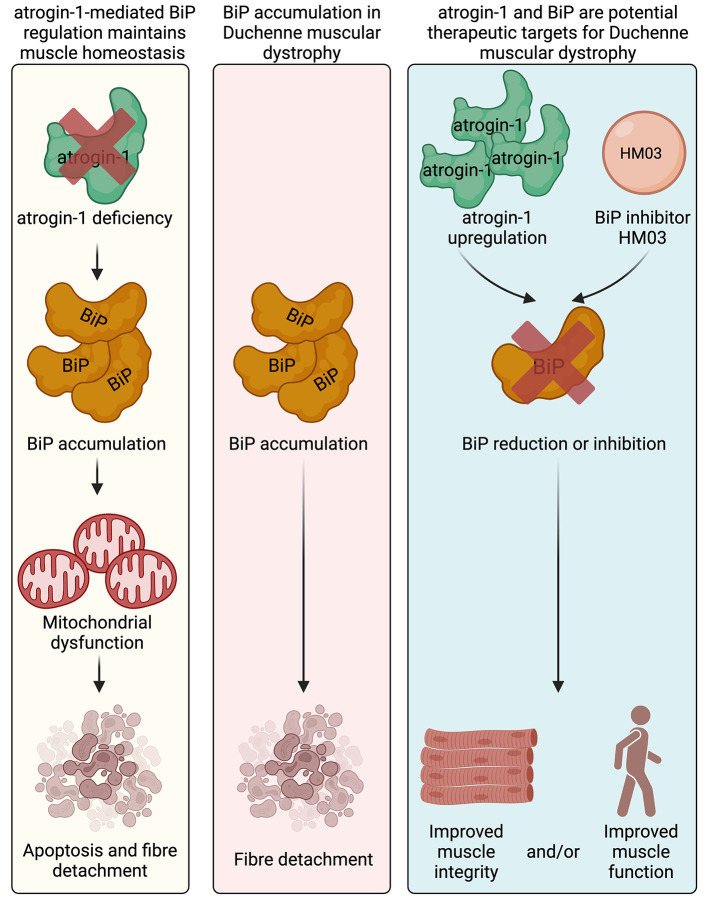

## Introduction

Skeletal muscle accounts for 30%–50% of human body mass, and it is not only indispensable for locomotion, but it also serves as a critical metabolic and storage organ (1). Consequently, loss of skeletal muscle mass — muscle atrophy — is characteristic of numerous clinical conditions and chronic diseases, including muscular dystrophies, sarcopenia, cancer cachexia, limb immobilization, bed rest, diabetes, congestive heart failure, and neurodegeneration. While muscle atrophy is a complex process with multifactorial etiology, a unifying feature in many of these pathologies is the upregulation of proteins of the ubiquitin-proteasome system, resulting in a shift in protein balance from net synthesis to net degradation and a subsequent loss in muscle mass. One such protein is atrogin-1 (also known as MAFbx and FBX032), a skeletal and cardiac muscle–specific E3 ubiquitin ligase that regulates the recognition of substrates, subsequently targeting them for ubiquitination (2, 3). Identified over 20 years ago, atrogin-1 is upregulated in a multitude of atrophy-inducing conditions (reviewed in ref. 4), not only promoting skeletal and cardiac muscle atrophy (2, 5, 6), but also inhibiting hypertrophy (7, 8). Consistent with this role, animals deficient in atrogin-1 are protected from skeletal muscle atrophy (2), although they present with myopathic phenotypes (9, 10), and cardiomyopathy (9, 11, 12) — highlighting a broader role of atrogin-1 in the maintenance of muscle mass and function. Indeed, atrogin-1 has recently been shown to mediate the interplay between the ubiquitin-proteasome system and autophagy, and a failure in this process results in apoptosis of cardiomyocytes and subsequent cardiomyopathy (12).

Given such a central role for atrogin-1 in regulating atrophy, and muscle homeostasis, a key focus over the past few years has been to identify its cellular targets. In line with this, binding studies and in vitro ubiquitin ligase assays have revealed multiple substrates of atrogin-1, including the myogenic regulatory factor MyoD (13); the eukaryotic translation initiation factor 3 subunit f (eIF3-f) (14); sarcomeric proteins myosin, vimentin, and desmin (15); and calcineurin (7), although most remain to be validated in vivo. In addition to this, mass spectrometry analyses comparing protein turnover rates between atrogin-1–KO and control cardiomyocytes identified the ESCRT-III protein CHMP2B as a target of atrogin-1 (12). Whether similar proteins are targeted in skeletal muscle remains unknown. There is, therefore, a need to identify the cellular targets of atrogin-1 in skeletal muscle and mechanistically dissect how atrogin-1 regulates skeletal muscle mass and homeostasis.

In the present study, we used the in vivo cell biology approaches afforded by the zebrafish model coupled with systems proteomics to examine the mechanism by which atrogin-1 regulates skeletal muscle mass during homeostasis and disease. We reveal that a loss in atrogin-1 resulted in an accumulation of the endoplasmic reticulum (ER) chaperone binding immunoglobulin protein (BiP), which leads to mitochondrial impairment and a subsequent loss in muscle fiber integrity, highlighting a mechanism by which atrogin-1 maintains muscle homeostasis. We further implicated atrogin-1–mediated BiP regulation in the pathogenesis of Duchenne muscular dystrophy (DMD) using a zebrafish model of the disease, thereby suggesting alternative avenues for therapeutic intervention in this and other muscle wasting disorders.

## Results

### Loss of atrogin-1 results in contraction-dependent fiber failure.

**Figure 1:**
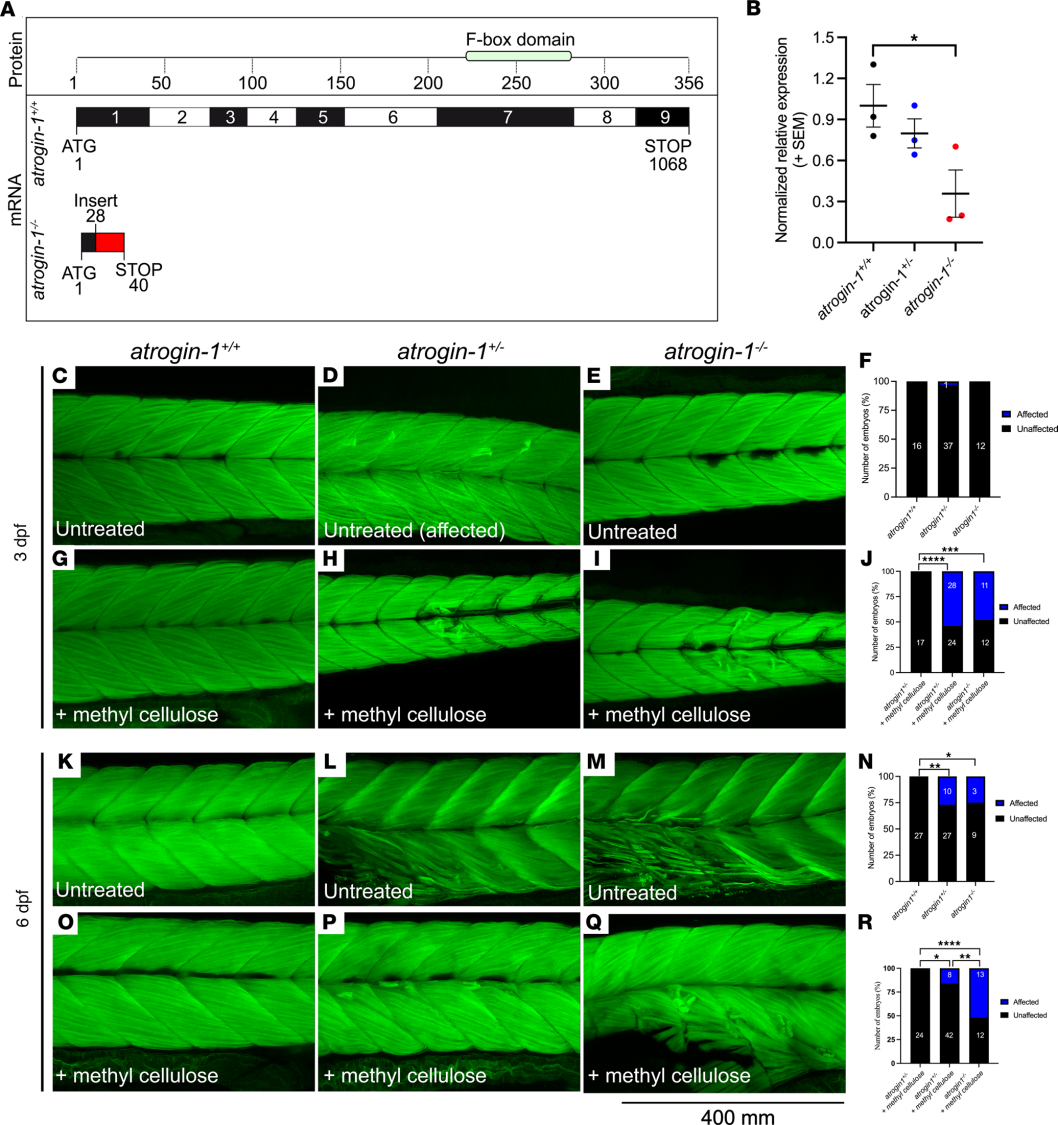
Atrogin-1 deficiency results in contraction-dependent muscle fiber detachment. Schematic of wild-type *atrogin-1* (*atrogin-1^+/+^*) and mutant *atrogin-1* (*atrogin-1^–/–^*) protein structure and mRNA sequence, with the mutant predicted to incorporate a premature stop in exon 1. The mutant was generated using CRISPR/Cas9 genome editing, which resulted in a 34 bp insertion (red). Numbers in the protein box are amino acids, and numbers in the mRNA box are base pairs. (**B**) qRT-PCR analysis showing significant reduction in *atrogin-1* levels in *atrogin-1^–/–^* mutants compared with *atrogin-1^+/+^* wild-type larvae. Error bars represent mean ± SEM for 3 replicate experiments, with each experiment comprising a pooled sample of at least 5 fish. *P < 0.05 determined using a 1-way ANOVA with Tukey’s multiple correction post hoc test. Muscle fibers span the entire length of the somite in the 3 dpf *atrogin-1^+/+^* (**C**), *atrogin-1* heterozygous (*atrogin-1*^+/–^) (**D**), and *atrogin-1^–/–^* mutant (**E**) larvae, as seen by F-Actin labeling. (**F**) Quantification of the muscle phenotype, with *atrogin-1^+/+^*, *atrogin-1*^+/–^, and *atrogin-1^–/–^* displaying indistinguishable muscle structure, as determined using a χ^2^ test. Incubation of 3 dpf *atrogin-1*^+/–^ (**H**) and *atrogin-1^–/–^* (**I**) in methyl cellulose results in muscle fiber detachment, which is not evident in *atrogin-1^+/+^* larvae (**G**). (**J**) Percentage of affected *atrogin-1^+/+^*, *atrogin-1*^+/–^, and *atrogin-1^–/–^* larvae, with the latter 2 genotypes having a significant increase in the proportion of fish displaying the muscle fiber detachment, as determined using a χ^2^ test. At 6 dpf, *atrogin-1*^+/–^ (**L**) and *atrogin-1^–/–^* (**M**) display sporadic muscle fiber detachment but not in *atrogin-1^+/+^* larvae (**K**). (**N**) Percentage of affected *atrogin-1^+/+^*, *atrogin-1*^+/–^, and *atrogin-1^–/–^* larvae, with the latter 2 genotypes having a significant increase in the proportion of fish displaying the muscle fiber detachment, as determined using a χ^2^ test. Methyl cellulose incubation of 6 dpf *atrogin-1*^+/–^ (**P**) and *atrogin-1^–/–^* (**Q**) results in muscle fiber detachment, which is not evident in *atrogin-1^+/+^* larvae (**O**). (**R**) Percentage of affected *atrogin-1^+/+^*, *atrogin-1*^+/–^, and *atrogin-1^–/–^* larvae, with the latter 2 genotypes having a significant increase in the proportion of fish displaying the muscle fiber detachment, as determined using a χ^2^ test. *P < 0.05, **P < 0.01, ***P < 0.001, ****P < 0.0001. All experiments were performed in triplicate, with the total number of fish examined in each replicate documented in Supplemental Table 2.

To determine the role of atrogin-1 in skeletal muscle, we used CRISPR/Cas9 genome editing to generate an atrogin-1 mutant (*atrogin-1^pc43/pc43^*, referred throughout to as *atrogin-1^–/–^*) with a 34–base pair insertion in exon 1, which resulted in a frameshift and incorporation of a premature stop codon (Figure 1A). Examination of *atrogin-1* mRNA levels revealed a significant reduction in *atrogin-1^–/–^* mutants compared with homozygous wild-type (*atrogin-1^+/+^*) sibling embryos (Figure 1B), suggestive of nonsense mediated decay of the mRNA. We next examined skeletal muscle integrity in the atrogin-1 mutants by staining the muscle with phalloidin, a high-affinity filamentous actin probe. While under normal conditions the muscle structure in *atrogin-1* heterozygous (*atrogin-1^+/–^*) and *atrogin-1^–/–^* mutants 3 days after fertilization (3 dpf) was indistinguishable from that of *atrogin-1^+/+^* embryos (Figure 1, C–F), *atrogin-1^+/–^* and *atrogin-1^–/–^* larvae displayed muscle fiber detachment following incubation in methyl cellulose, a viscous solution that increases the load on the muscle (Figure 1, G–J). Additionally, at 6 dpf, both *atrogin-1^+/–^* and *atrogin-1^–/–^* larvae displayed sporadic muscle fiber detachment (Figure 1, K–N), which was exacerbated following incubation in methyl cellulose (Figure 1, N–R). To validate that the observed phenotype was indeed due to a deficiency in atrogin-1, we compared the pathology evident in a second atrogin-1 mutant allele (*atrogin-1^pc44/pc44^*) previously generated using zinc finger nuclease technology. Examination of the muscle in 6 dpf *atrogin-1^pc44/pc44^* mutants revealed presence of detached fibers identical to the phenotype seen in the *atrogin-1^–/–^* mutants (Supplemental Figure 1, A–D; supplemental material available online with this article; https://doi.org/10.1172/jci.insight.167578DS1). As further evidence that atrogin-1 deficiency is responsible for the fiber detachment phenotype seen in the mutant, we performed a rescue experiment whereby we overexpressed fluorescently tagged actin (Lifeact-GFP) or fluorescently tagged atrogin-1 (atrogin-1-GFP) in the muscle of *atrogin-1^–/–^* mutants and examined their ability to prevent muscle fiber disintegration. As presented in Supplemental Figure 1, E–G, while 38% (23 of 60 fibers in 6 larvae) of Lifeact-GFP–expressing muscle fibers underwent detachment, atrogin-1-GFP expression (22 fibers in 6 larvae) was sufficient to prevent fiber disintegration. Taken together, a reduction in or a loss of atrogin-1 resulted in load-dependent muscle detachment.

### Atrogin-1 deficiency results in impaired membrane integrity and apoptosis.

**Figure 2:**
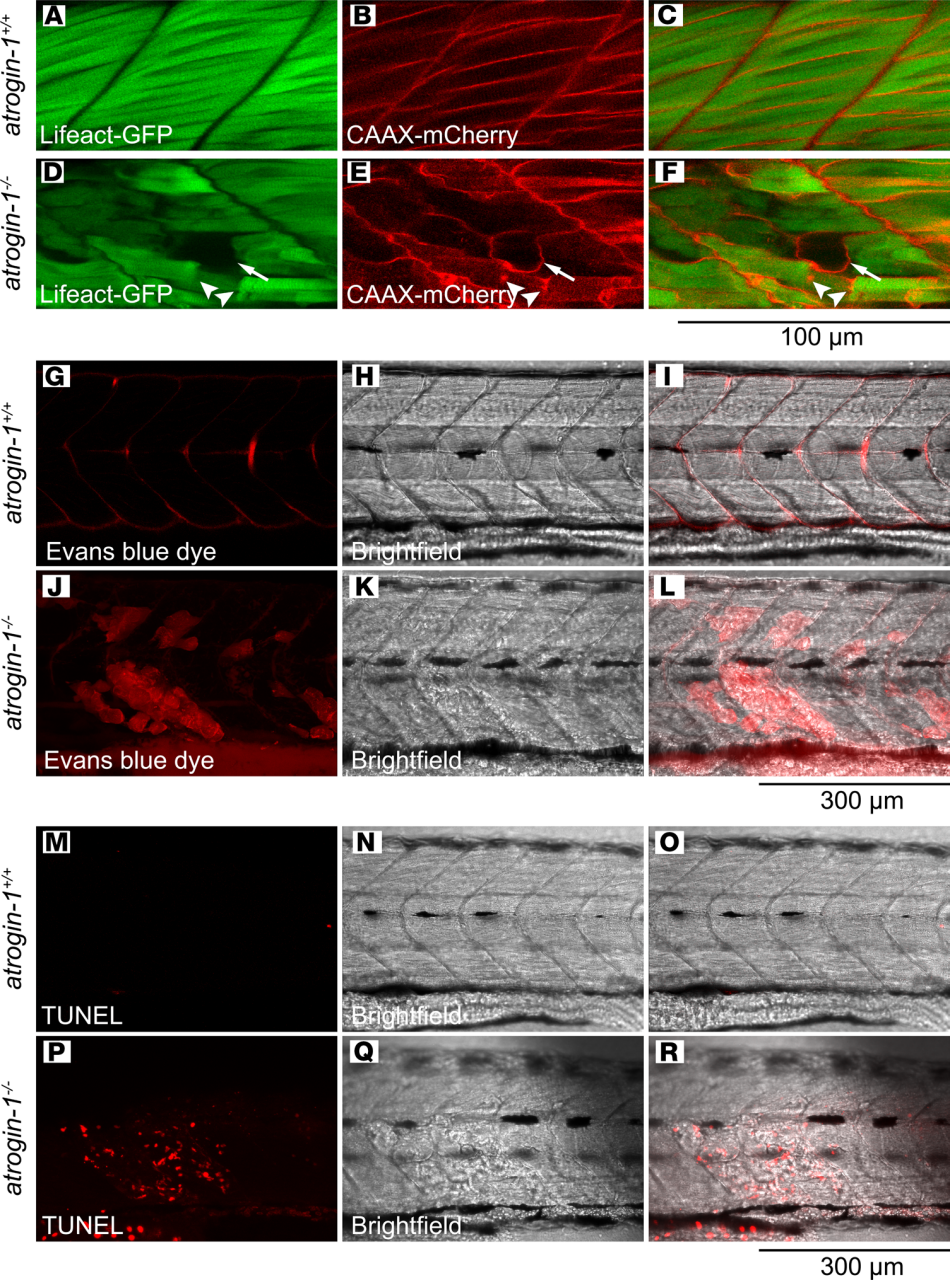
Atrogin-1 deficiency results in impaired membrane integrity and apoptosis. Live images of methyl cellulose–treated 6 dpf *atrogin-1^+/+^* (**A**–**C**) and *atrogin-1^–/–^* (**D**–**F**) on the (Tg(*actc1b*:Lifeact-GFP);Tg(*actc1b*:CAAX-mCherry) background, whereby the actin filaments within the muscle fibers are labeled with GFP and membrane and t-tubules with mCherry. While muscle cells in the *atrogin-1^+/+^* wild-type larvae span the entire length of the somite, with the sarcolemma fully surrounding each cell, *atrogin-1^–/–^* mutants display detached muscle fibers that are surrounded with irregular sections of sarcolemma, along with presence of abnormal sarcolemma vacuoles. Live images of methyl cellulose–treated 6 dpf *atrogin-1^+/+^* (**G**–**I**) and *atrogin-1^–/–^* (**J**–**L**) larvae injected with Evans blue dye. *atrogin-1^–/–^* mutants displayed an accumulation of the dye, which is absent in *atrogin-1^+/+^* larvae. TUNEL staining on 3 dpf methyl cellulose–treated *atrogin-1^+/+^* (**M**–**O**) and *atrogin-1^–/–^* (**P**–**R**) larvae, with the latter displaying TUNEL in areas where the muscle cell had detached. Scale bar: 100 μm (**A**–**F**); 300 μm (**G**–**R**).

We next examined the biological basis of the atrogin-1 mutant muscle detachment phenotype by crossing it to a double transgenic line (Tg(*actc1b*:Lifeact-GFP);Tg(*actc1b*:CAAX-mCherry). In this line actin filaments within the muscle fibers are labeled with GFP, and membrane and t-tubules with mCherry. In methyl cellulose–treated 6 dpf wild-type sibling larvae, muscle cells span the entire length of the somite, with the sarcolemma fully surrounding each cell (Figure 2, A–C). In stark contrast, in *atrogin-1^–/–^* mutants, detached muscle fibers were surrounded with irregular sections of sarcolemma, with abnormal sarcolemma vacuoles also evident (Figure 2, D–F), indicative of a deficit in sarcolemma integrity. To test sarcolemmal permeability, we performed precardiac sinus injections of Evans blue dye, a small-molecular-weight dye that while impermeable in cells with normal sarcolemma, selectively accumulates in cells in which the sarcolemma lacks integrity. Using this technique, we revealed that while muscle fibers in *atrogin-1^+/+^* larvae had no Evans blue dye uptake (Figure 2, G–I), consistent with the presence of intact sarcolemma, muscle cells in *atrogin-1^–/–^* mutants displayed an accumulation of dye (Figure 2, J–L), confirming an impairment in membrane integrity. Finally, to determine if the retracted muscle cells seen in the *atrogin-1^–/–^* mutant undergo apoptosis, we performed a TUNEL assay in methyl cellulose–treated, 6 dpf *atrogin-1^+/+^* and *atrogin-1^–/–^* mutants. In line with the normal muscle structure observed in *atrogin-1^+/+^* wild-type larvae, we observed no apparent TUNEL labeling (Figure 2, M–O). In contrast, *atrogin-1^–/–^* mutants displayed increased numbers of TUNEL^+^ nuclei, which coincided with areas of muscle detachment (Figure 2, P–R). Collectively, these results highlight that loss of atrogin-1 results in a loss in membrane integrity and increased apoptosis, consistent with the phenotypes presented in atrogin-1–deficient cardiomyocytes.

### Untargeted proteomics identified a role of atrogin-1 in regulating BiP levels.

**Figure 3:**
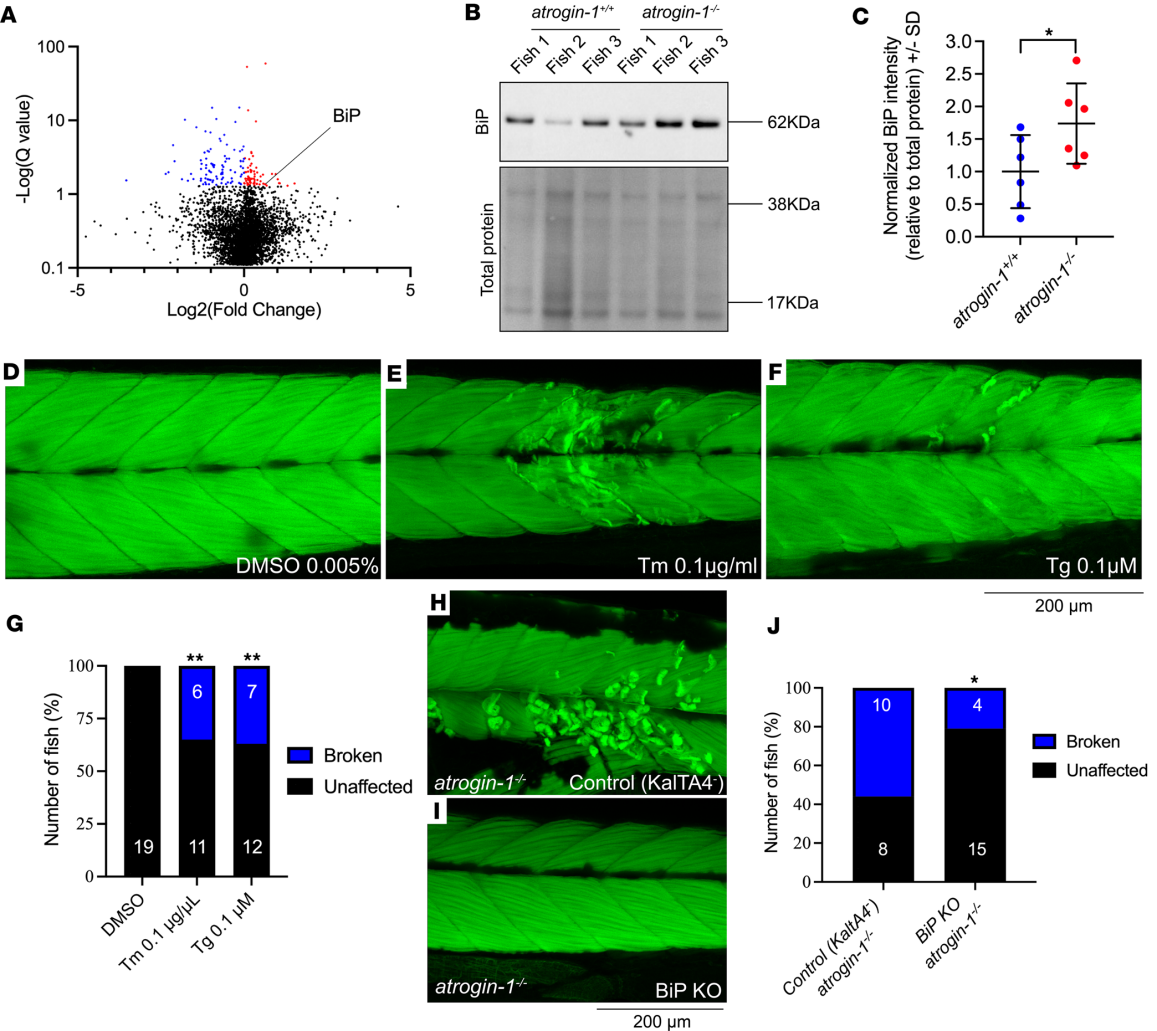
Atrogin-1 mutants display increased levels of BiP, which is sufficient to cause muscle fiber detachment. (**A**) Volcano plot highlighting differentially regulated proteins in *atrogin-1^–/–^* larvae compared with *atrogin-1^+/+^* wild-type larvae – identified from untargeted proteomics. Proteins significantly (*q* < 0.05) upregulated and downregulated are shown in red and blue, respectively, as determined using an unpaired *t* test. (**B**) Representative Western blot images for BiP, and total protein direct blue stain, on whole cell protein lysates obtained from 3 independent biological replicates, each containing multiple *atrogin-1^+/+^* or *atrogin-1^–/–^* larvae. (**C**) Quantification of BiP levels normalized to total protein with *atrogin-1^–/–^* larvae displaying a significant reduction compared with *atrogin-1^+/+^*, as determined using an unpaired *t* test. Data are shown as mean ± SD. (**D**–**F**) 6 dpf tunicamycin- (Tm-) or thapsigargin-treated (Tg-treated) larvae display muscle fiber detachment following incubation in methyl cellulose. (**G**) The percentage of affected larvae, with Tm or Tg treatment resulting in a significant increase in the proportion of fish displaying the muscle fiber detachment, as determined using a χ^2^ test. (**H** and **I**) Confocal images of F-actin–stained, methyl cellulose–treated, 6 dpf *atrogin-1^–/–^* mutants on the Tg(*actc1b*:KalTA4;*cryaa*:GFP^pc54Tg^) only [labeled as Control (KaltA4)] or Tg(*actc1b*:KalTA4;*cryaa*:GFP^pc54Tg^) and Tg(4XUAS:NLSCas9;*cmlc2*:RFP ^gl37Tg^) (labeled as BiP KO) background. While control *atrogin-1^–/–^* mutants display fiber detachment, *atrogin-1^–/–^* mutants with BiP deficiency specifically in the muscle show normal muscle structure. (**J**) The percentage of affected *atrogin-1^–/–^* control larvae and BiP-KO larvae, with the latter having a significant decrease in the proportion of fish displaying the muscle fiber detachment, as determined using Fisher’s exact test. *P < 0.05, **P < 0.01. All experiments performed in triplicate with the total number of fish examined in each replicate being documented in Supplemental Table 2. Scale bar: 200 μm.

Having characterized the atrogin-1 mutant phenotype, we wanted to examine the mechanisms by which loss of atrogin-1 results in fiber detachment. Given the striking similarity in phenotypes between the zebrafish atrogin-1–KO animals and the atrogin-1–KO cardiomyocytes, we hypothesized that the same protein(s) may be dysregulated in both systems following the absence of atrogin-1, resulting in the pathology observed. To identify proteins differentially regulated in the zebrafish mutant, we performed mass spectrometry on protein lysates obtained from 6 dpf *atrogin-1^+/+^* and *atrogin-1^–/–^* larvae. Using this strategy, we identified a total of 4,242 distinct proteins across the 6 samples (Supporting Data Values file). Of these, 162 proteins were differentially expressed in the *atrogin-1^–/–^* mutant larvae compared with *atrogin-1^+/+^* larvae (Figure 3A), of which, 69 were upregulated and 93 downregulated (Supporting Data Values file). Given that a loss in atrogin-1 is expected to result in an accumulation of its targets — as they are no longer targeted for degradation — we focused on proteins that were upregulated in the mutant. A comparison of the 69 proteins upregulated in the *atrogin-1^–/–^* mutant with those shown to also have increased expression (56 proteins) or reduced turnover (137 proteins) in the atrogin-1–KO cardiomyocytes (12) revealed an overlap of 7 proteins (Table 1). Of these 7 proteins, the most upregulated was BiP (also known as GRP-78 or heat shock 70 kDa protein 5 [HSPA5]), a member of the HSP70 family of proteins localized primarily to the ER, where it regulates multiple processes, including activation of the unfolded protein response (UPR) following accumulation of unfolded or misfolded proteins, protein transport, cell survival and apoptosis, calcium homeostasis, and ER-mitochondrial calcium crosstalk, which subsequently regulates mitochondrial function (16, 17) (reviewed in ref. 18). Importantly, prolonged ER stress and chronic upregulation of BiP results in apoptosis, which is a characteristic feature of the atrogin-1 mutant, and atrogin-1–deficient cardiomyocytes (12). BiP accumulation is therefore a prime candidate that could explain the manifestation of atrogin-1 mutant phenotype, and as such all subsequent analyses were focused on defining BiP’s role in maintaining muscle homeostasis.

To confirm that BiP is indeed upregulated in the atrogin-1 mutant, we performed Western blotting for BiP on whole cell protein lysate. In line with our untargeted proteomics data, *atrogin-1^–/–^* larvae displayed a significant increase in BiP compared with *atrogin-1^+/+^* larvae (Figure 3, B and C). One possible explanation for the increased levels of BiP is increased transcription. To test this possibility, we performed qRT-PCR for the UPR genes *bip*, *chop*, *atf6*, and *atf4*. Our results indicate no difference in the expression of these genes between *atrogin-1^+/+^* wild-type and *atrogin-1^–/–^* larvae (Supplemental Figure 2A). Therefore, the increased levels of BiP seen in the mutant were not due to increased transcription. We next wished to determine if BiP is a direct target of atrogin-1, which could explain the increased levels of BiP in the atrogin-1 mutant. To this end, we conducted coimmunoprecipitation experiments in HEK293T cells by cotransfecting with plasmids encoding GFP (control), Myc-tagged zebrafish atrogin-1, and/or HA-tagged zebrafish BiP and, subsequently, performing a pull-down assay using anti-Myc–coated beads. While Myc-atrogin-1 was enriched in the Myc-atrogin-1 and in the Myc-atrogin-1– and BiP-HA–transfected cells, indicating successful pull down, no HA-tagged BiP was detected in any of the immunoprecipitated lysates (Supplemental Figure 2B). These results indicate that BiP is not a direct target of atrogin-1. Our results suggest that BiP levels are indirectly regulated by atrogin-1 and the increased abundance in the atrogin-1 mutant is likely a secondary consequence of atrogin-1 deficiency.

### Accumulation of BiP results in muscle detachment following the loss of atrogin-1.

Having confirmed that BiP is upregulated in atrogin-1–deficient larvae, we next wanted to determine if its increased level was responsible for the loss in muscle integrity observed in the *atrogin-1^–/–^* mutant. To address this, we treated 3 dpf larvae with the well-characterized ER stress inducers tunicamycin (Tm) or thapsigargin (Tg) for 3 days, which not only induced *bip* expression, but also expression of the UPR genes *chop*, *atf6*, and *atf4* (Supplemental Figure 2C). Examination of muscle structure in Tm- or Tg-treated fish revealed a significant increase in the proportion of muscle fibers displaying muscle fiber detachment following incubation in methyl cellulose (Figure 3, D–G), consistent with the morphology seen in the *atrogin-1^–/–^* mutants. Therefore, chronic ER activation of ER stress can explain the characteristic phenotype evident in the *atrogin-1^–/–^* mutant.

To more explicitly implicate BiP accumulation as the mechanism responsible for the muscle fiber detachment seen in the *atrogin-1^–/–^* mutant, we made use of the compound HM03, which has been shown to selectively inhibit BiP activity by binding to its substrate binding domain (19). We treated 3 dpf *atrogin-1^–/–^* mutants with the BiP inhibitor HM03 or DMSO for 3 days, changing the chemical each day thereafter, and at 6 dpf we examined muscle integrity. Although the rescue was not complete, pharmacological inhibition of BiP resulted in a reduction in the number of *atrogin-1^–/–^* mutants displaying fiber disintegration (Supplemental Figure 2, D–F), supporting the role of BiP accumulation in driving the myopathic phenotype seen following atrogin-1 deficiency.

As an alternative approach, we attempted to generate a BiP mutant line using CRISPR/Cas9 genome editing. However, despite using low levels of BiP targeting guide RNAs, BiP crispant larvae displayed striking phenotypes, including edema in the brain and heart (Supplemental Figure 3, A and B) that were lethal and thus limited our ability to recover BiP germline mutants. Indeed, mice that are completely deficient in BiP display peri-implantation lethality (20). To overcome this issue, we developed a muscle-specific BiP-KO strategy to examine its ability to rescue the *atrogin-1^–/–^* mutant phenotype. Briefly, this involves the use of two transgenic lines on the *atrogin-1^–/–^* mutant background: a muscle-specific KalTA4 expressing line (Tg(*actc1b*:KalTA4;cryaa:GFP^pc54Tg^) crossed to a UAS-driven Cas9 line (Tg(4XUAS:NLSCas9;cmlc2:RFP ^gl37Tg^) (Supplemental Figure 3C). Into this line, we injected two BiP-targeting guide RNAs at the 1-cell stage, which is predicted to result in muscle-specific mutagenesis of BiP (BiP KO) and subsequent loss in expression. To confirm the loss of BiP expression, we stained 3 dpf BiP-KO fish with antibody against myosin and BiP. While 89% of control fish, which were injected with the dual BiP gRNAs but lack KalTA4, displayed striated ER-like BiP localization, only 27% of BiP-KO fish showed clear striations, with the remaining 73% lacking this staining pattern (Supplemental Figure 3, D–F), confirming the loss of BiP expression. To further support this, examination of BiP levels using Western blot on whole cell lysates demonstrated a significant reduction of BiP in BiP-KO fish compared with control fish (Supplemental Figure 3, G and H). Collectively, these results confirm that our tissue-specific approach results in a reduction in BiP expression in the muscle.

Having confirmed the efficiency of our tissue-specific KO approach, we examined the muscle morphology in 6 dpf BiP-KO, *atrogin-1^–/–^* mutant larvae. Remarkably, muscle-specific KO of BiP resulted in a striking rescue of the fiber integrity defects of *atrogin-1^–/–^* mutant larvae (Figure 3, H–J). While 56% of *atrogin-1^–/–^* mutants displayed detached muscle fibers, this was significantly reduced to 21% following muscle-specific loss of BiP in *atrogin-1^–/–^* mutants (Figure 3J). A potential explanation for this rescue is that muscle-specific loss of BiP results in reduced muscle contraction, thus preventing fiber detachment. To exclude this possibility, we examined locomotor function, using the Zebrabox assay — which examines the average distance, time, and, thus, speed a fish moves over a 10-minute period — of 6 dpf, BiP-KO larvae. As shown in Supplemental Figure 3I, the average speed traveled by BiP-KO larvae is indistinguishable from that of control larvae, highlighting that muscle-specific loss of BiP does not affect motor performance. Taken together, these results highlight a role of atrogin-1–mediated BiP regulation in the maintenance of muscle homeostasis.

### Systems proteomics reveals impaired mitochondrial dynamics as the mechanism of muscle fiber detachment in atrogin-1–deficient fish.

**Figure 4:**
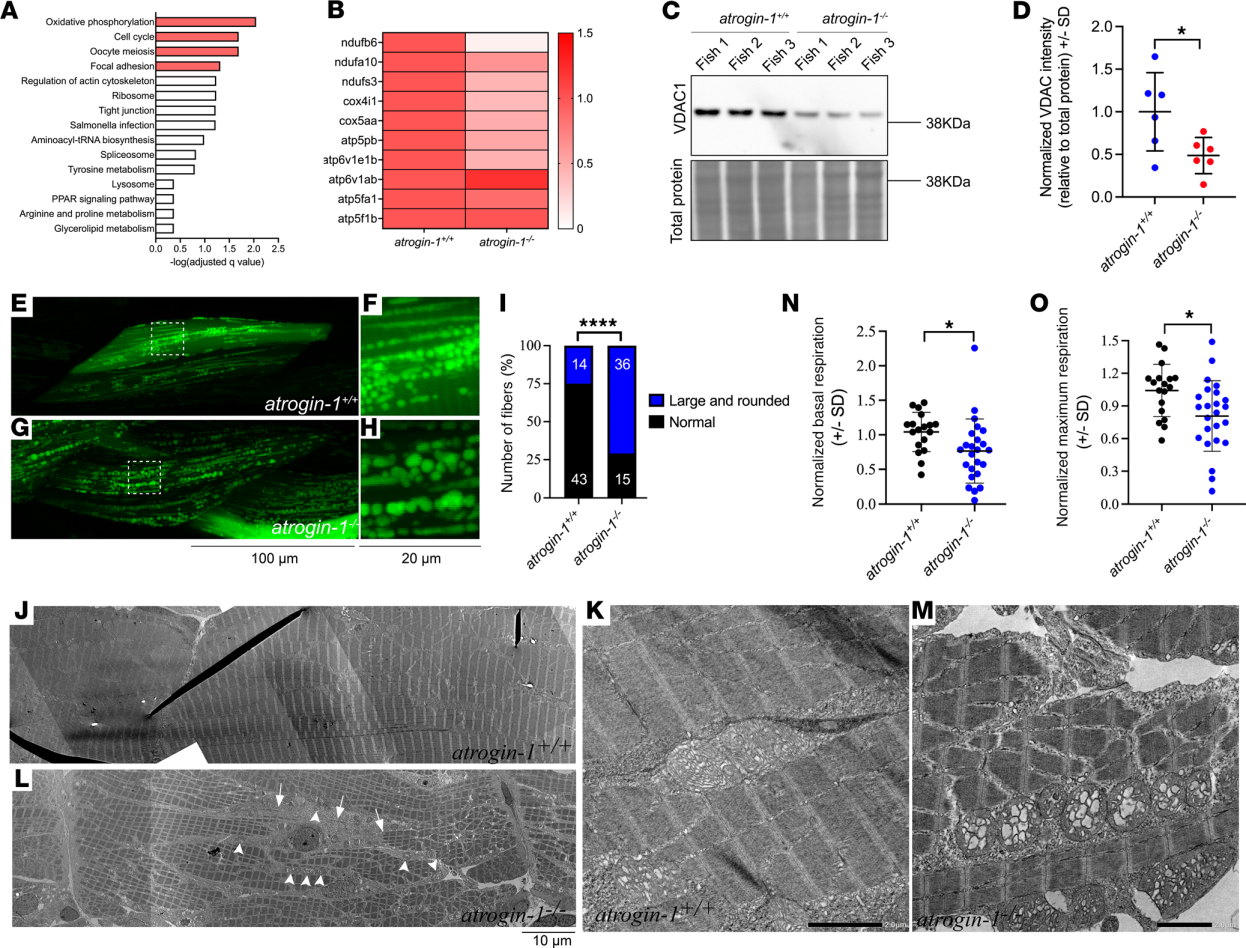
Atrogin-1 deficiency also results in altered mitochondrial dynamics. (**A**) Overrepresentation analyses on all differentially regulated proteins in *atrogin-1^–/–^* larvae revealed a significant enrichment of multiple Kyoto Encyclopedia of Genes and Genomes pathway terms, with oxidative phosphorylation (OXPHOS) being the most significant. (**B**) Heatmap of the relative abundance of OXPHOS proteins ndufb6, ndufa10, ndufs3, cox4i1, cox5aa, atp5pb, atp6v1e1b, atp6v1ab, atp5fa1, and atp5f1b in *atrogin-1^+/+^* and *atrogin-1^–/–^* larvae. (**C**) Representative Western blot images for VDAC1, and total protein direct blue stain, on whole cell protein lysates obtained from 3 independent biological replicates, each containing multiple *atrogin-1^+/+^* or *atrogin-1^–/–^* larvae. (**D**) Quantification of VDAC1 levels normalized to total protein, with *atrogin-1^–/p4^* larvae displaying a significant reduction compared with *atrogin-1^+/+^* larvae, as determined using an unpaired *t* test. Data are shown as mean ± SD. (**E**–**H**) Live images of 6 dpf methyl cellulose–treated *atrogin-1^+/+^* (**E** and **F**) and *atrogin-1^–/–^* (**G** and **H**) larvae showing mosaic expression of *actc1b*:mitoGFP labeling the mitochondria in green. While *atrogin-1^+/+^* larvae display small mitochondria some of which form an intricate network, mitochondria in *atrogin-1^–/–^* larvae are large and rounded. Scale bar: 100 μm (left); 20 μm (right). **F** and **H** are zoomed in views of **E** and **G**, respectively. (**I**) The proportion of muscle fibers displaying altered mitochondrial morphology in methyl cellulose–treated *atrogin-1^+/+^* or *atrogin-1^–/–^* larvae, as per Fisher’s exact test. ****P < 0.0001. (**J**–**M**) Electron micrographs of the muscle in 6 dpf methyl cellulose–treated *atrogin-1^+/+^* and *atrogin-1^–/–^* larvae. While *atrogin-1^+/+^* larvae display normal sarcomeric and mitochondrial structure (**J** and **K**), *atrogin-1^–/–^* mutants (**L** and **M**) display fiber disintegration, evident by the disorganized arrangement of sarcomeres (arrow), and abnormal mitochondria with large and swollen matrices (arrowheads). **K** and **M** are zoomed in views of **J** and **L**, respectively. (**N** and **O**) 3 dpf methyl cellulose–treated *atrogin-1^–/–^* larvae show a significant reduction in basal (**N**) and maximum respiration (**O**) compared with *atrogin-1^+/+^* larvae, as determined using an unpaired *t* test. Data are shown as mean ± SD.*P < 0.05; ****P< 0.0001. All experiments performed in triplicate, with the total number of fish examined in each replicate being documented in Supplemental Table 2.

While our results support a model in which BiP accumulation results in fiber detachment in atrogin-1 mutants, we wished to determine how this was regulated at a cellular level. To this end, we reexamined our atrogin-1 mutant proteomics data set to identify any potential pathways that may be dysregulated in the mutant. Enrichment analyses on all differentially regulated proteins in *atrogin-1^–/–^* larvae revealed a significant overrepresentation of proteins of the oxidative phosphorylation (OXPHOS) pathway (Figure 4A), which is responsible for the production of ATP in the mitochondria. Further examination of proteins within this Kyoto Encyclopedia of Genes and Genomes (KEGG) pathway revealed that except for atp6v1ab and atp5f1b, all other proteins (ndufb6, ndufa10, ndufs3, cox4i1, cox5aa, atp5pb, atp6v1e1b, and atp5fa1) were downregulated in *atrogin-1^–/–^* larvae compared with *atrogin-1^+/+^* larvae (Figure 4B). One possible explanation for this is the reduced transcription of each of these complexes. However, our qRT-PCR analyses revealed small, nonsignificant increases in expression of each of these genes, highlighting that reduced transcription is not responsible for the reduction in OXPHOS abundance observed in *atrogin-1*–deficient larvae (Supplemental Figure 4A). An alternative explanation for the changes in OXPHOS levels is a change in mitochondrial fission and fusion rates and the subsequent reduction in mitochondria number. To assess mitochondrial fusion and fission, we examined the expression of mitochondrial fission genes *drp1* and *fis1* and fusion genes *mfn1*, *mfn2*, and *opa1*. With the exception of *opa1*, all genes examined were significantly downregulated in the atrogin-1 mutant, suggestive of altered mitochondrial dynamics (Supplemental Figure 4, B and C). We also examined total mitochondrial content, using Western blot for VDAC1 on whole cell protein lysate, and, in line with our hypothesis, *atrogin-1^–/–^* larvae displayed a significant reduction in VDAC1 levels compared with *atrogin-1^+/+^* larvae, indicating a reduction in mitochondrial content (Figure 4, C and D).

Having identified altered mitochondrial biology in the atrogin-1 mutant, we wished to further characterize their mitochondrial structure and function. Mitochondrial morphology was examined by expressing a mito-GFP construct (generated by fusing the mitochondrial targeting sequence of Cox8a to GFP) specifically in the muscle of methyl cellulose–treated *atrogin-1^+/+^* wild-type and *atrogin-1^–/–^* mutant larvae. Live imaging at 6 dpf revealed that while the majority of muscle fibers in methyl cellulose–treated *atrogin-1^+/+^* fish display small mitochondria, some of which form an intricate network (Figure 4, E, F, and I), a significant proportion of muscle fibers in methyl cellulose–treated *atrogin-1^–/–^* mutant larvae displayed large and rounded mitochondria (Figure 4, G–I). Consistent with this, electron microscopy revealed that 6 dpf methyl cellulose–treated *atrogin-1^–/–^* mutants displayed fiber disintegration, evident by the disorganized arrangement of sarcomeres and abnormal mitochondria with large and swollen matrices (Figure 4, L, M, and O), with methyl cellulose–treated *atrogin-1^+/+^* larvae displaying normal sarcomeric and mitochondrial structure (Figure 4, J, K, and N). Finally, oxygen consumption rates, a readout of mitochondrial function, were also examined in the *atrogin-1*–deficient larvae. We report a significant reduction in both basal (Figure 4N) and maximum respiration (Figure 4O) in 3 dpf methyl cellulose–treated *atrogin-1^–/–^* mutant larvae, indicating an alteration in mitochondrial function. Collectively, these results highlight that loss of atrogin-1 results in a reduction in mitochondria number and an impairment in mitochondrial structure and function.

We next wished to determine if the mitochondrial alterations observed could explain the muscle fiber detachment phenotype seen in *atrogin-1^–/–^* mutant larvae. To this end, we treated 3 dpf wild-type larvae with rotenone, a complex 1 inhibitor, for 3 days and examined the muscle using an antibody against F-actin. Remarkably, while DMSO treatment had no effect on muscle integrity (Supplemental Figure 4D), chronic inhibition of mitochondrial function resulted in muscle fiber detachment (Supplemental Figure 4, E and F), identical to the phenotype seen in *atrogin-1^–/–^* mutants. These results highlight that impaired mitochondrial dynamics is sufficient to cause muscle fiber detachment.

### BiP accumulation is responsible for impaired mitochondrial biology.

**Figure 5:**
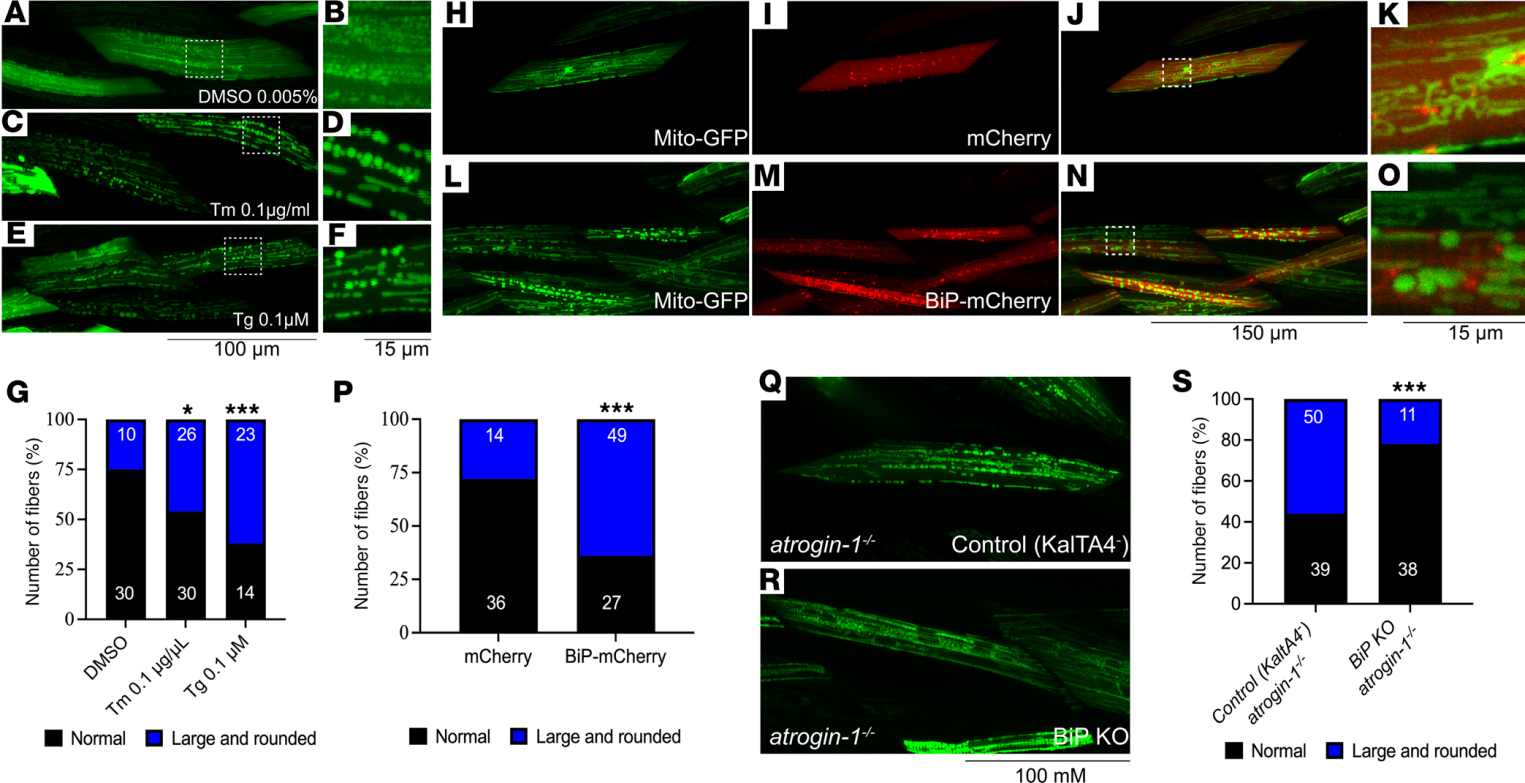
BiP accumulation is also responsible for the impaired mitochondrial dynamics. Live images of 6 dpf DMSO- (**A** and **B**), tunicamycin- (Tm-) (**C** and **D**), or thapsigargin-treated (Tg-treated) (**E** and **F**) larvae showing mosaic expression of *actc1b*:mitoGFP labeling the mitochondria in green. While DMSO-treated larvae display small mitochondria, some of which form an intricate network, mitochondria in Tm- and Tg-treated larvae were large and rounded, as determined using a χ^2^ test. **B**, **D**, and **F** (scale bar: 15 μm) are zoomed in views of **A**, **C**, and **E** (scale bar: 100 μm), respectively. (**G**) The proportion of muscle fibers displaying altered mitochondrial morphology following DMSO, Tm, or Tg treatment, determined using a χ^2^ test. (**H**–**O**) Live images of 6 dpf larvae coexpressing Mito-GFP with mCherry (**H**–**K**) or BiP-mCherry (**L**–**O**). **K** and **O** are zoomed in views of **J** and **N**, respectively. Scale bar: 150 μm (first, second, and third columns); 15 μm (last column). (**P**) The proportion of muscle fibers displaying altered mitochondrial morphology comparing mCherry overexpression with BiP overexpression, as per Fisher’s exact test. (**Q** and **R**) Live images of 6 dpf *atrogin-1^–/–^* mutant larvae on the Tg(*actc1b*:KalTA4;cryaa:GFP^pc54Tg^) only (labeled as Control (KaltA4)) or Tg(*actc1b*:KalTA4;cryaa:GFP^pc54Tg^) and Tg(4XUAS:NLSCas9;cmlc2:RFP ^gl37Tg^) (labeled as BiP KO) background, showing mosaic expression of *actc1b*:mitoGFP labeling the mitochondria in green. While control *atrogin-1^–/–^* mutants display large and rounded mitochondria, *atrogin-1^–/–^* mutants with BiP deficiency specifically in the muscle have small mitochondria that form an intricate network, as determined using a χ^2^ test. Scale bar: 100 μm. (**S**) The proportion of muscle fibers displaying altered mitochondrial morphology in control and BiP-KO *atrogin-1^–/–^* mutants, as per Fisher’s exact test. *P < 0.05, ***P < 0.001. All experiments performed in triplicate with the total number of fish examined in each replicate being documented in Supplemental Table 2.

To determine if the mitochondrial phenotypes seen in the atrogin-1 mutant are caused by BiP accumulation, we treated 3 dpf larvae expressing the mito-GFP transgene with Tm or Tg for 3 days and examined mitochondrial morphology. Our analyses revealed that chronic treatment with Tm or Tg resulted in a significant increase in the proportion of muscle fibers displaying large and rounded mitochondria compared with those in F-actin DMSO-treated animals (Figure 5, A–G), consistent with the morphology seen in the *atrogin-1^–/–^* mutants. To more explicitly implicate BiP accumulation as the mechanism responsible for the mitochondrial phenotypes seen in the atrogin-1 mutant, we generated a construct to enable the muscle-specific overexpression of fluorescently tagged, full-length mouse BiP. To confirm that the fluorescently tagged form of BiP localized correctly to the ER, we stained BiP-mCherry–expressing fish with an anti-mCherry antibody and with an antibody against Ryr1, which is known to localize within the t-tubule. Using super resolution imaging, BiP-mCherry was found to localize to the terminal cristae of the sarcoplasmic reticulum (SR), a structure directly adjacent to the T-tubules, and more generally within the SR network (Supplemental Figure 5, A–C). Having confirmed that fluorescent tagging of BiP does not affect its localization, we coinjected the BiP-mCherry construct (or mCherry alone) along with the mitochondria labeling GFP plasmid to examine the effect of BiP overexpression on mitochondrial structure. Remarkably, while mCherry-expressing muscle cells displayed small, intricate mitochondrial networks (Figure 4I and Figure 5, H–K), BiP-mCherry–expressing fibers had predominantly large and rounded mitochondria that phenocopied the *atrogin1* loss-of-function phenotype (Figure 5, L–O and P). This demonstrates that BiP upregulation alone is sufficient to cause the abnormal mitochondrial structure observed in the *atrogin-1*–deficient fish.

As a final approach, we used our muscle-specific BiP-KO system to examine if loss of BiP is sufficient to rescue the mitochondrial phenotype seen in the atrogin-1 mutant. Indeed, while 56% of muscle fibers in *atrogin-1^–/–^* mutant larvae contained large and rounded mitochondria, this was significantly reduced to 22% in BiP muscle–specific KO, *atrogin-1^–/–^* mutants, highlighting a rescue in the mitochondrial phenotypes (Figure 5, Q–S).

We next wished to determine if BiP overexpression altered mitochondrial dynamics as seen in the *atrogin-1*^–/–^ mutant. To this end, wild-type embryos were injected with mCherry or BiP-mCherry RNA, and at 2 dpf qRT-PCR for UPR and mitochondrial fission and fusion genes was performed. Consistent with the injection of RNA, BiP-mCherry–injected fish displayed increased levels of BiP (Supplemental Figure 5D). Interestingly, we also observed a significant increase in the expression of *atf4*, with *chop* showing a small nonsignificant increase, suggesting that BiP overexpression may have triggered ER stress (Supplemental Figure 5D). Furthermore, in line with the reduced expression of mitochondrial fission and fusion genes observed in *atrogin-1*^–/–^ mutants, BiP-mCherry RNA–injected larvae displayed a significant reduction in *drp1*, *fis1*, *mfn2*, and *opa1*, with *mfn1* showing small but nonsignificant reduction (Supplemental Figure 5, E and F), highlighting a role of BiP in regulation mitochondrial dynamics.

Taken together, our results demonstrate that the loss of atrogin-1 results in the accumulation of BiP, which results in mitochondrial dysfunction and a subsequent detachment and apoptosis of muscle cells.

### Atrogin-1 is a modifier in, and contributes to, the pathogenesis of DMD.

**Figure 6:**
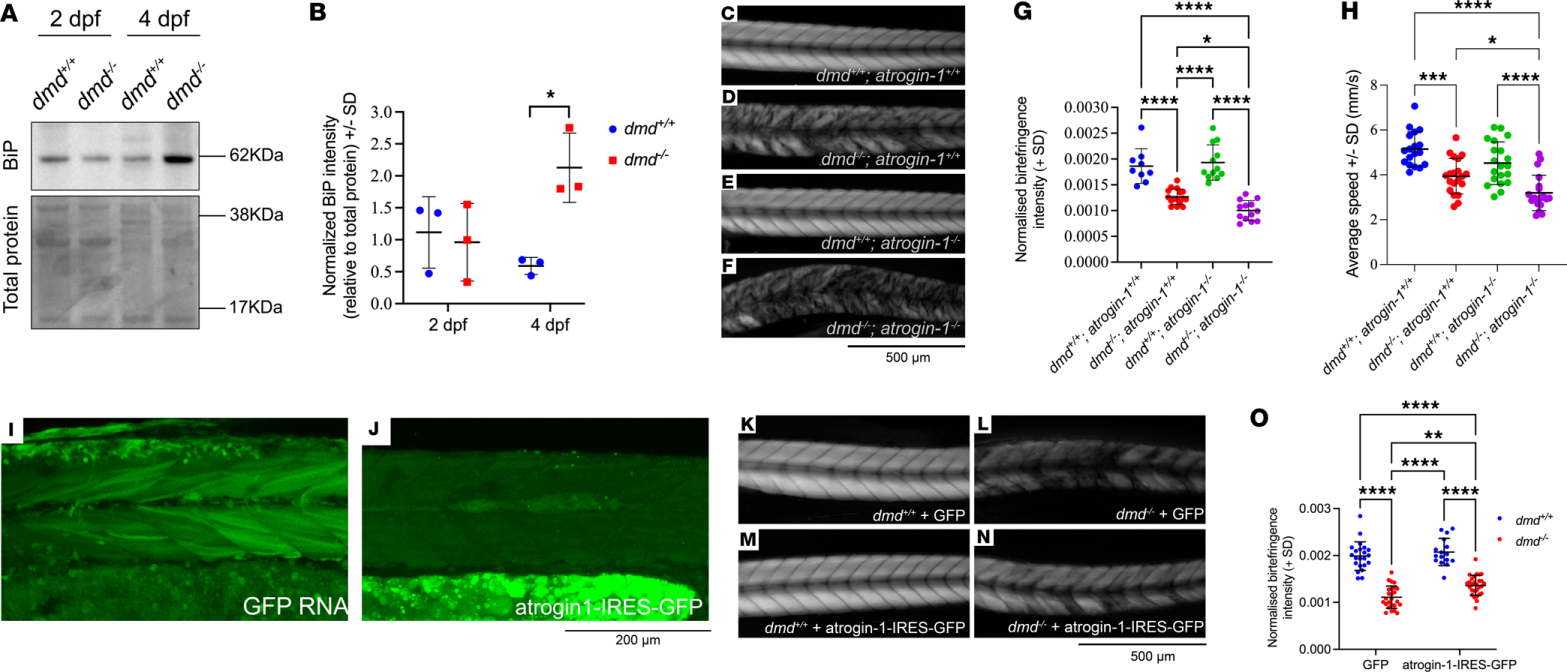
Atrogin-1 is a modifier of and contributes to the pathogenesis of Duchenne muscular dystrophy. (**A**) Representative Western blot image for BiP, and total protein direct blue stain, on whole cell protein lysates obtained from 2 dpf and 4 dpf *dmd^+/+^* or *dmd^–/–^* larvae. (**B**) Quantification of BiP levels normalized to total protein with 4 dpf *dmd^–/–^* larvae displaying a significant increase compared with *dmd^+/+^* larvae, as determined using a 2-way ANOVA with Šidák’s multiple correction post hoc test. Data are shown as mean ± SD. (**C**–**F**) Representative birefringence images of 4 dpf *dmd^+/+^; atrogin-1*^+/+^ wild-type larvae and *dmd^–/–^ and atrogin-1^–/–^* single and double mutants. Scale bar: 500 μm. (**G**) Quantification of normalized birefringence intensity, which is the mean birefringence intensity relative to area, in 4 dpf *dmd^+/+^; atrogin-1*^+/+^ wild-type larvae and *dmd^–/–^ and atrogin-1^–/–^* single and double mutants, analyzed using a 1-way ANOVA with Tukey’s multiple correction post hoc test. Data are shown as mean ± SD. (**H**) Average speed, in mm/s, of *dmd^+/+^; atrogin-1*^+/+^ wild-type larvae and *dmd^–/–^ and atrogin-1^–/–^* single and double mutants, as analyzed using as a 1-way ANOVA with Tukey’s multiple correction post hoc test. Data are shown as mean ± SEM for 3–4 biological replicates. (**I** and **J**) Live images of 4 dpf larvae expressing *GFP* RNA or *atrogin-1-IRES-GFP* RNA. Scale bar: 200 μm. (**K**–**N**) Birefringence images of 4 dpf *dmd^+/+^* and *dmd^–/–^* larvae injected with *GFP* RNA or *atrogin-1-IRES-GFP* RNA. Scale bar: 500 μm. (**O**) Quantification of normalized birefringence intensity, which is the mean birefringence intensity relative to area, in 4 dpf *dmd^+/+^* or *dmd^–/–^* larvae injected with *GFP* RNA or *atrogin-1-IRES-GFP* RNA, as analyzed using a 2-way ANOVA with Šidák’s multiple correction post hoc test. Data are shown as mean ± SD. *P < 0.05, **P < 0.01, ***P < 0.001, ****P < 0.0001. All experiments performed in triplicate with the total number of fish examined in each replicate being documented in Supplemental Table 2.

The muscle fiber detachment observed in the *atrogin-1^–/–^* mutant is strikingly similar to the phenotype seen in zebrafish models of DMD, caused by a mutation in *dystrophin* (21). Given that our findings have implicated BiP accumulation in the presentation of the *atrogin-1^–/–^* mutant phenotype, we hypothesized that a similar mechanism may be contributing to the pathogenesis of DMD. Indeed, BiP upregulation has been reported in several mammalian models of DMD (22, 23), but whether a similar response occurs in zebrafish is not known. To determine this, we performed Western blotting for BiP on whole cells lysates of 2 dpf and 4 dpf *dmd^+/+^* wild-type and *dmd^–/–^* mutant larvae. Consistent with the mammalian models, we observed a significant increase in BiP expression in 4 dpf *dmd^–/–^* mutant compared with the *dmd^+/+^* wild-type larvae, although no change was observed at 2 dpf (Figure 6, A and B). To determine if loss of *dystrophin* results in increased ER stress and activation of the UPR, we performed qRT-PCR for the UPR genes *bip*, *chop*, *atf6*, and *atf4*. We observed a significant increase in the expression of *bip* and *atf6*, with *chop* and *atf4* showing small but nonsignificant increases (Supplemental Figure 6A). Therefore, as shown in the *mdx* mouse model, and in skeletal muscle from patients with DMD (22–24), the loss of dystrophin in zebrafish also results in increased abundance of BiP and activation of the UPR.

Having confirmed that BiP is upregulated in zebrafish models of DMD, we wished to determine if the atrogin1-BiP axis we have identified contributes to DMD pathology and could be manipulated for potential therapeutic gain. As such, we crossed the *dmd^–/–^* mutant with the *atrogin-1^–/–^* mutant and examined muscle structure, using birefringence assays and locomotor function in the double mutants. As previously shown, *dmd^–/–^* mutants displayed a significant reduction in mean birefringence intensities compared with wild-type larvae (Figure 6, C, D, and G), highlighting a reduction in muscle fiber integrity. The birefringence intensities in *atrogin-1^–/–^* mutants on the other hand were indistinguishable from those of wild-type larvae (Figure 6, C, E, and G), consistent with the mild, sporadic phenotypes seen in these mutants. Simultaneous loss of both dystrophin and atrogin-1 resulted in a dramatic additive reduction in birefringence intensity within the myotomes of double mutant larvae, compared not only with wild-type and *atrogin-1^–/–^* mutants, but also *dmd^–/–^* mutants (Figure 6, C, F, and G). This highlights a potential role of atrogin-1 in modifying the muscle fiber detachment in DMD. We also examined if loss of atrogin-1 affects muscle function in *dmd^–/–^* mutants, specifically examining the average speed of larvae over a 10-minute period in a standard zebrabox locomotion assay (25). Similar to the birefringence assays, while *dmd^–/–^* mutants have a significant reduction in average speed, it is further reduced following the loss of atrogin-1 (Figure 6H). The exacerbation of the muscle detachment phenotype and reduction in muscle function in *dmd^–/–^* mutants following the loss of atrogin-1 demonstrates a role of the latter in DMD pathogenesis.

As further validation of atrogin-1’s role in DMD, we injected *atrogin-1-IRES-GFP* (or *GFP* control) RNA in *dmd^–/–^* mutants and examined muscle fiber integrity in 4-day-old animals. Expression of RNA was confirmed by the fluorescence of GFP protein in the myotome of injected larvae (Figure 6, I and J). *atrogin-1-IRES-GFP* mRNA injection significantly ameliorated the reduction in birefringence intensity evident in *dmd^–/–^* larvae, although the rescue was not complete — that is, *atrogin-1-IRES-GFP*–injected *dmd^–/–^* mutants still has a significant reduction in birefringence compared with *dmd^+/+^* wild-type larvae injected with same RNA (Figure 6, K–O). This is a surprising finding, because it suggests that in DMD additional dystrophin-independent mechanisms regulated by atrogin-1 may be contributing to disease pathogenesis. Importantly, atrogin-1 overexpression did not have any detrimental effect on muscle integrity, as evident by indistinguishable birefringence intensities between GFP-injected and *atrogin-1-IRES-GFP* injected wild-type larvae (Figure 6, K–O). These results combined with the data on the *dmd^–/–^; atrogin-1^–/–^* double mutants implicates atrogin-1 in the presentation of DMD pathologies.

### BiP inhibition rescues muscle function in DMD.

**Figure 7:**
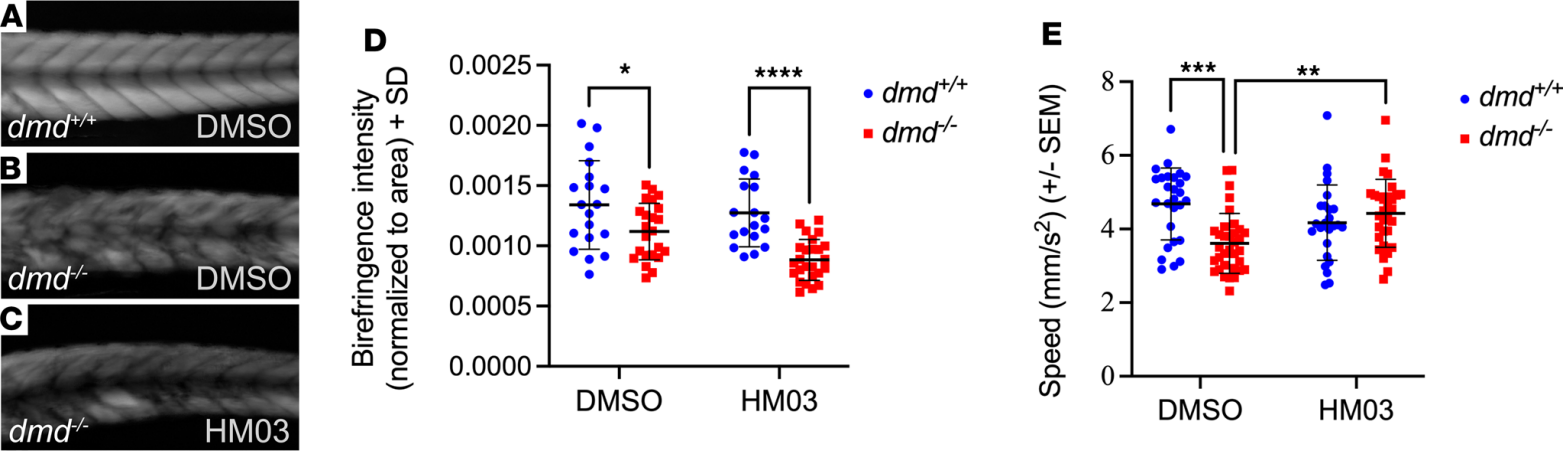
BiP inhibition rescues muscle function in DMD. (**A**–**C**) Representative birefringence images of 6 dpf DMSO-treated *dmd^+/+^* and *dmd^–/–^* larvae and HM03-treated *dmd^–/–^* larvae. (**D**) Quantification of normalized birefringence intensity, which is the mean birefringence intensity relative to area, in 6 dpf DMSO- or HM03-treated *dmd^+/+^* and *dmd^–/–^* larvae, as analyzed using a 2-way ANOVA with Šidák’s multiple correction post hoc test. Data are shown as mean ± SD. (**E**) Average speed, in mm/s, of 6 dpf DMSO- or HM03-treated *dmd^+/+^* and *dmd^–/–^* larvae. Data are shown as mean ± SEM for 3–4 biological replicates and were analyzed using a 2-way ANOVA with Šidák’s multiple correction post hoc test. *P < 0.05, **P < 0.01, ***P < 0.001, ****P < 0.0001. All experiments performed in triplicate with the total number of fish examined in each replicate being documented in Supplemental Table 2.

While our results suggest that atrogin-1 may be manipulated for therapeutic gain in DMD, we wanted to examine whether manipulating levels of BiP, which is regulated by atrogin-1, could provide a possible alternative therapeutic strategy to combat DMD. To test this, we treated 3 dpf *dmd^+/+^* wild-type larvae and *dmd^–/–^* mutants with the BiP inhibitor HM03, or DMSO control for 3 days, changing the chemical each day thereafter, and at 6 dpf we performed muscle integrity birefringence assays and zebrabox assays. Contrary to our hypothesis, HM03 treatment had no effect on muscle integrity, evident from the indistinguishable birefringence intensities between DMSO-treated and HM03-treated *dmd^–/–^* mutants (Figure 7, A–D). We confirmed this result by treating *dmd^–/–^* mutants on the Tg(*actc1b*:Lifeact-GFP);Tg(*actc1b*:CAAX-mCherry) background, whereby the actin filaments within the muscle fibers were labeled with GFP and membrane and t-tubules with mCherry (Supplemental Figure 6, B–D). Similar to the birefringence assays, HM03-treated *dmd^–/–^* mutants displayed similar severities of fiber detachment to DMSO-treated *dmd^–/–^* mutants. While these results are surprising, they suggest that rescue of muscle fiber integrity in the *dmd^–/–^* following atrogin-1 overexpression likely results from an atrogin-1 target independent of BiP.

We also examined the effect of HM03 treatment and subsequent BiP inhibition on the muscle function of *dmd^–/–^* mutants. Unlike the results for muscle integrity, HM03 treatment significantly improved the average speed of *dmd^–/–^* mutants compared with DMSO-treated *dmd^–/–^* mutants (Figure 7E). Remarkably, the mean speed of HM03-treated *dmd^–/–^* mutants was comparable to that of DMSO-treated *dmd^+/+^* wild-type larvae, indicating that HM03 completely restored muscle function in the mutant fish (Figure 7E). Importantly, treatment of *dmd^+/+^* wild-type larvae with HM03 had no effect on their speed, demonstrating that the improvement in muscle function seen in *dmd^–/–^* mutants was specific and not a generalized response.

Together, these results demonstrated that HM03 and the subsequent inhibition of BiP specifically improves muscle function in performance in *dmd^–/–^* mutants. Therefore, while the atrogin-1–mediated BiP may not be involved in the loss in fiber integrity seen in DMD, it does contribute to the reduction in muscle function, making this disease axis therapeutically relevant for potentially improving muscle performance in boys with DMD.

## Discussion

In the current study, we characterized the skeletal muscle of zebrafish deficient in atrogin-1, an E3 ubiquitin ligase that is upregulated in numerous muscle wasting conditions. We reveal that the loss of atrogin-1 resulted in the detachment and apoptosis of skeletal muscle fibers — consistent with the myopathic phenotypes seen following the transient knockdown of atrogin-1 — and displayed striking defects in mitochondrial structure and function (9). Using a systems proteomics approach, we further reveal that these phenotypes are attributed to the accumulation of BiP, the master regulator of ER/SR, which results in impaired mitochondrial dynamics and a subsequent detachment of muscle fibers. Atrogin-1 is therefore not only important in regulating catabolic processes but may also be indirectly regulating broader ER and mitochondrial regulated processes critical for the maintenance of muscle fiber integrity. It is noteworthy that, while the current study focused on characterizing this function of atrogin-1, our proteomics data set also identified some of the previously characterized targets of atrogin-1, including myosin and desmin (15), to be differentially regulated. Therefore, in addition to interacting with and regulating levels of sarcomeric proteins, and other proteins such as transcription factors and proteins of the ubiquitin proteasome system and autophagy/lysosome system, our study provides evidence of atrogin-1’s involvement in regulating ER-related cellular processes. Additionally, while there was a clear overlap in differentially regulated proteins identified in skeletal muscle lacking atrogin-1 (this study) and in cardiomyocytes deficient in atrogin-1 (12), there were also proteins that were unique to each tissue. This suggests that atrogin-1 and potentially other E3 ubiquitin ligases have specific targets that may differ across different tissues. Collectively, our results highlight a requirement of atrogin-1 in maintaining muscle homeostasis and suggest that its inhibition may result in dysregulation of ER- and mitochondrial-related processes. This is particularly relevant to therapies that aim to ameliorate muscle wasting by inhibiting atrogin-1. Our results suggest that, while short-term atrogin-1 inhibition may be beneficial, chronic inhibition is expected to be deleterious, resulting in ER defects, mitochondrial impairment, and a loss in muscle fiber integrity. Indeed, atrogin-1 mutations have recently been shown to cause dilated cardiomyopathy due to the upregulation of ER-stress–mediated apoptosis (26), supporting the unsuitability of atrogin-1 inhibition in treating muscle wasting.

The muscle fiber detachment observed following the loss of atrogin-1 is strikingly similar to the phenotype seen in zebrafish models of DMD (21), caused by a mutation in *dystrophin,* suggesting that atrogin-1 and BiP may contribute to the pathogenesis of the disease. Contrary to findings of previous studies, which showed amelioration of DMD pathology in zebrafish using the ubiquitin-proteasome system inhibitor MG132 (27), we showed that loss of atrogin-1 exacerbates the DMD phenotype and that upregulation can rescue muscle pathology. The latter is a highly surprising finding, because it suggests that in DMD additional dystrophin-independent mechanisms may be contributing to muscle fiber detachment, which can be ameliorated by increasing atrogin-1 levels, thus providing alternative therapeutic approaches for DMD. Consistent with this idea, cardiomyocyte-specific upregulation of atrogin-1 has been shown to reduce collagen deposition and fibrosis in the aged heart (28). Further studies to identify specific inducers of atrogin-1 and their use in treating DMD and potentially other muscle diseases are therefore needed.

In addition to targeting atrogin-1, our work has revealed BiP inhibition as an alternative strategy for the treatment of DMD. We observed a striking increase in BiP in the zebrafish DMD model, which is consistent with the upregulation seen in mammalian models of DMD (22, 23). Previous studies have shown that increased levels of BiP and of ER stress in general result in an impairment in the crosstalk between the SR and mitochondria, which subsequently disrupts calcium handing (22). Based on this, we propose that BiP accumulation has similar consequences in skeletal muscle of dystrophin-deficient zebrafish and that the specific inhibition of BiP may provide an alternative therapeutic strategy. To test this, we used the compound HM03, identified from cascade in silico screening, to specifically inhibit BiP by binding to its substrate binding domain and subsequently inhibiting cancer and tumour cell viability (19). Consistent with this improvement in a cancer setting, the specific inhibition of BiP using HM03 resulted in significant rescue in muscle function in dystrophin-deficient zebrafish. One caveat is that the locomotory assays utilized are also a read-out of neuromuscular function. Therefore, the improvements in muscle function noted following HM03 treatment could also be due to changes in other nonautonomous systems, such as motor neuron function. Importantly, while BiP inhibitors have previously not been tested in DMD, treatment of mdx mice with the ER chaperone tauroursodeoxycholic acid (TUDCA) has been shown to reduce ER stress and restore the SR-mitochondria interaction and calcium dynamics, subsequently improving contractile function. Furthermore, KO of caspase-12, a downstream effector of the UPR in *mdx* mice restores muscle force, emphasizing the role of ER stress in DMD pathology (23). It is noteworthy that, while atrogin-1 overexpression resulted in an unexpected improvement in muscle pathology, HM03 had no effect. This discrepancy could be explained by the incomplete inhibition of BiP by HM03 treatment, which is supported by the lack of severe BiP crispant-like phenotypes in the HM03-treated fish. An alternative explanation is that the rescue in muscle fiber integrity seen in dystrophin-deficient zebrafish following atrogin-1 overexpression likely results from an atrogin-1–mediated mechanism independent of BiP. In any case, the ability to fully rescue muscle function using HM03 suggests that BiP is the primary contributor to the reduction in muscle function in DMD and provides a more specific target than the previously tested TUDCA for improving muscle function in DMD. Upregulation of BiP and ER stress/UPR–related factors is also observed in several other types of muscle diseases (reviewed in ref. 29), including limb-girdle muscular dystrophy caused by mutations in FKRP (30, 31) and caveolin 3 (32), sporadic inclusion body myositis caused by mutations in GNE (33), and tibial muscular dystrophy caused by mutations encoding Titin (34). Inhibition of BiP using HM03 or alternative approaches may therefore also be relevant for the treatment of these disorders.

In conclusion, we have identified a role of atrogin-1–mediated BiP regulation in the pathogenies of DMD. While the inhibition of BiP resulted in a striking rescue in the muscle function, it had no effect on muscle pathology. This is a remarkable finding because it highlights that the reduction in muscle function and loss in muscle fiber integrity are caused by different cellular processes. As such, while corrective gene therapies to restore dystrophin expression and subsequently correct muscle attachment lie on the horizon, deficits in muscle contractile and metabolic function may persist even after successful transduction. Approaches, such as those highlighted in the current study, including atrogin-1 upregulation or BiP inhibition, will likely be needed to complement gene-based interventions for the treatment of DMD and other muscle wasting disorders.

## Methods

### Sex as a biological variable.

Given that sex is undetermined in zebrafish larvae, sex was not considered as a biological variable.

### Fish maintenance.

Briefly, adult zebrafish were maintained in recirculating aquarium systems and were kept between 26°C and 28°C on a 14-hour-light/10-hour-dark cycle. Fish were differentially fed according to their age: larvae (up to 10 dpf) were fed paramecia twice daily; larvae (10–14 dpf) were fed paramecia twice daily and a single feed of *Artemia salina*; juveniles (10–30 dpf) were fed *Artemia salina* twice daily; and fish aged 30 days and older were fed *Artemia salina* twice daily and appropriate sized pellets. Animals were group housed in 3 L tanks, with a maximum of 6 fish per liter. The health of the fish was monitored on a daily basis. All experiments were carried out on embryos of TU/TL background. Fish were anesthetized using Tricaine methanesulfonate (3-amino benzoic acidethylester; MilliporeSigma, E10521) at a final concentration of 0.16% in E3 embryo medium (5 mM NaCl, 0.17 mM KCl, 0.33 mM CaCl, 0.33 mM MgSO4, 0.00004% [v/v] methylene blue in water, pH 7.2). To exacerbate the loss in muscle integrity in *atrogin-1^–/–^* mutants, larvae were incubated in 1% methyl cellulose for 2 hours, as per refs. 35, 36, and subsequently fixed and processed for immunofluorescence.

### Generation of plasmids and transgenic and mutant strains.

Vectors for transgenesis were generated using the Tol2kit system (37). Briefly, 3 entry clones (5′, middle, and 3′) were recombined into a fourth destination vector backbone. The muscle-specific mito-GFP construct (*actc1b*:mito-GFP) was generated using p5E-*actc1b* (38), pME-mitoGFP (this study), and p3E-pA and pDEST-Tol2-pA2 (37). *Actc1b*-KalTA4 (39) was also made using p5E-*actc1b* and p3E-pA but used pME-KalTA4 (39) as the middle entry clone and a modified destination vector that had a GFP lens reporter (pDEST-Tol2pA2-*aCry*-EGFP) (40). The atrogin-1-IRES-GFP plasmid was generated with *p5E-CMV/*SP6 (37), full-length zebrafish atrogin-1 in the middle entry position (pME-*atrogin-1*; this study), and IRES-GFP in the 3′ position (37). pME-mitoGFP contains the first 31 amino acids of the zebrafish cytochrome *c* oxidase subunit 8A (cox8a) sequence fused to GFP (41). This vector was produced by subcloning the mito-GFP sequence from the original MLS-EGFP vector (41) into pDONR221; primers are included in Supplemental Table 3. pME-atrogin-1 was cloned by amplifying full-length zebrafish atrogin-1 (primers are included in Supplemental Table 3), which was subsequently cloned into pDONR221. Plasmids were injected at 30 ng/μL into 1-cell-stage embryos along with transposase RNA (25 ng/μL) that was synthesized from the pcs2FA-transposase vector (40) using the mMessage machine Sp6 kit (Ambion, AM1340). Plasmids for transfection were synthesized by Genscript. Myc-atrogin-1 was generated by subcloning the full-length zebrafish atrogin-1 sequence in the pcDNA3.1(+)-N-Myc backbone using the Kpn1/BamHI cloning site; BiP-HA was generated by subcloning the full-length zebrafish Bip coding sequence into the pcDNA3.1(+)-C-HA backbone using BamHI/Apa1 cloning site.

The *atrogin-1* mutant strain (*atrogin-1^–/–^*) was generated using the CRISPR/Cas9 system. Synthetic gRNAs targeting *atrogin-1* were generated as crRNA:tracrRNA duplexes (Alt-R CRISPR–Cas9 system, IDT). The following *atrogin-1* targeting crRNA sequence, identified using the ZiFIT program, with PAM sequences in uppercase letters, was used: 5′-ggacaagactggcggtctccTGG-3′. The *atrogin-1* targeting crRNA was heteroduplexed to universal tracrRNA according to the manufacturer’s protocol to generate bipartite gRNAs, which was subsequently was injected into one-cell-stage embryos, along with Cas9 protein and Cascade Blue dye (Molecular Probes, D1976). Primers used for the generation and genotyping of the *atrogin-1^–/–^* mutant strain are listed in Supplemental Table 3. An additional atrogin-1 mutant strain (*atrogin-1^pc44/pc44^*) was generated using Zinc-finger nuclease technology. mRNAs encoding a pair of Zinc-finger nucleases targeted to exon 4 of the atrogin-1 locus (CompoZr Knockout Zinc Finger Nuclease, MilliporeSigma) were injected into single-celled zebrafish embryos at a final concentration of 50 ng/μL. Allele-specific PCR KASP technology (Geneworks) was used for *atrogin-1^pc44/pc44^* genotyping. For tissue-specific KO of BiP, bipartite BiP targeting gRNA sequences were designed using the IDT Alt-R CRISPR-Cas9 guide RNA tool. Specificity was examined in silico, and sequences that had high on-target and low off-target scores were selected. Specificity was also examined using the BLAST tool, and gRNA sequences that matched BiP with high confidence (low E value) and no other loci were selected. To achieve muscle-specific knockdown of BiP, Tg(*actc1b*:KalTA4;*cryaa*:GFP^pc54Tg^) was crossed to Tg(4XUAS:NLSCas9;cmlc2:RFP ^gl37Tg^) (each line on the *atrogin-1^–/–^* mutant background), and in the resulting embryos, 2 BiP targeting bipartite gRNAs (listed in Supplemental Table 1) were injected at the 1-cell stage. At 3 dpf, the fish were sorted for green lens and red hearts to confirm the presence of *cryaa*:GFP and *cmlc2*:RFP linked to *actc1b*: KalTA4 and *actc1b*:4XUAS:NLSCas9, respectively. Existing mutant and transgenic lines used include: *dmd^pc2/pc2^*(21), Tg(*actc1b*:mCherry-CAAX)^–2Tg^ (42), Tg(*actc1b*:lifeact-GFP)^-1Tg^ (42), and Tg(4×UAS:NLS-cas9, *cmlc2*:RFP)^gl37Tg^ (43). With the exception of electron microscopy experiments, seahorse assays, and HM03 drug rescue on the atrogin-1 mutant, heterozygous adults were crossed to generate a pool of homozygous wild-type and heterozygous and homozygous mutant larvae, and different parents were used for each replicate. For electron microscopy, seahorse assays, and HM03 drug rescue experiments on the atrogin-1 mutant, *atrogin-1* wild-type or mutant adults were crossed to obtain aged-matched larvae for each of the experiments, and different parents were used for each replicate.

### Immunofluorescence and quantitative RT-PCR.

Zebrafish immunofluorescence experiments were performed according to previously published protocols (44). The following antibodies/vital dyes were used in this study: Rhodamine-tagged phalloidin (Molecular Probes, 1:200), anti-BiP (MilliporeSigma, G9043, 1:500), and anti-Ryr1 (MilliporeSigma, R129, 1:100). The primary antibody was washed at least 6 times with PBST, following which the samples were incubated in appropriate secondary antibodies: Alexa Fluor–labeled 488, Alexa Fluor–labeled 546, and/or Alexa Fluor–labeled 596 (Invitrogen, 1:300). Stained embryos were mounted in 1% low-melting-point agarose and imaged using a Zeiss LSM 710 confocal microscope (phalloidin and TUNEL stains, Evans Blue Dye labeling) or Zeiss LSM 980 confocal microscope with super-resolution Airyscan 2 (BiP stained). The maximum intensity projections were obtained using Fiji (http://fiji.sc). The phenotype was scored prior to performing genotyping assays to ensure the researcher was blinded to the genotype.

### cDNA synthesis and quantitative RT‑PCR.

Total RNA was extracted using TRI Reagent (MilliporeSigma). cDNA was synthesized by Superscript III Reverse Transcriptase (Invitrogen Life Technologies). Quantitative RT-PCR (qRT-PCR) was performed using a Lightcycler (Roche) using SYBR Green Master mix (Roche). Primers used for RT-PCR analysis are listed in Supplemental Table 3.

### RNA synthesis.

For *atrogin-1* RNA rescue experiments, GFP or atrogin-1-IRES-GFP RNA was synthesized using the mMessage mMachine SP6 Transcription Kit (Ambion). RNA was injected at a concentration of 50 ng/μL into 1-cell-stage embryos. Injected embryos were sorted for GFP labeling prior to analysis.

### TUNEL staining.

6 dpf methyl cellulose–treated fish were fixed in 4% paraformaldehyde (PFA) for 2 hours at room temperature. The fixed fish were washed twice with PBST (137 mM NaCl, 2.7 mM KCl, 10 mM Na_2_HPO4, 1.8 mM KH_2_PO4 and 0.1% [w:v] Tween 20 [MilliporeSigma Aldrich, P9416]) and then stored overnight in 100% methanol at –20°C. On the next day, embryos were incubated in precooled 100% acetone for 10 minutes at –20°C, following which they were washed with PBST 3 times, for 10 minutes each time. The embryos were then permeabilized with of 0.1% Triton X-100 and 0.1% sodium citrate in PBS for 15 minutes. TUNEL staining was then performed as per manufacturer’s protocol (In Situ Cell Death Detection Kit, POD, Roche, 11684817910). To achieve a brighter signal, TUNEL-stained embryos were blocked in blocking solution (10% fetal calf serum, 0.1% Tween 20, and 1% DMSO in PBS) for 1 hour and stained with an anti-fluorescein antibody (Roche, 1426346910, 1:2000) overnight at 4^o^C. The next day, the embryos were washed 8 times in PBST and stained with an appropriate secondary antibody (Alexa Fluor 488 donkey anti-sheep antibody,Life Technologies, A11015, 1:300) overnight at 4^o^C. Finally, the embryos were washed 8 times in PBST and imaged using a Zeiss LSM 710 confocal microscope.

### Evans blue dye injections.

A working injection mix (0.1% Evans blue dye) was diluted using Ringer’s solution prior to injection of 5 nL into the pericardial vein of 6 dpf larvae obtained from a heterozygous incross. After injection, zebrafish embryos were placed in Ringer’s solution and incubated in the dark at 28°C for 3 hours, following which they were incubated in 1% methyl cellulose for 2 hours to induce fiber damage. Larvae were subsequently mounted in 1% low-melting-point agarose and imaged using a Zeiss LSM 710 confocal microscope.

### Electron microscopy.

6 dpf zebrafish larvae were fixed in 2.5% glutaraldehyde, 2% paraformaldehyde in 0.1 M sodium cacodylate buffer, pH 7.4, overnight at 4°C. After washing in 0.1 M sodium cacodylate tissue was post-fixed in 2% osmium tetroxide in 0.1 M sodium cacodylate, rinsed in distilled water, and then dehydrated in acetone and embedded in Epon using a Pelco Biowave Pro. After polymerization, ultrathin sections (90 nm) were cut using an Ultracut Leica Ultramicrotome and stained with 2% uranyl acetate in distilled water and lead citrate. The sections were imaged with a Jeol 1400Plus TEM.

### Cell culture and transfection.

HEK293T cells were grown in DMEM supplemented with 1% antibiotics and 10 % fetal bovine serum. About 14 μg of vectors containing the constructs were used to transfect the cells using the Lipofectamine 3000 (Gibco, Thermo Fisher Scientific, L3000001) following the manufacturer’s instructions. Briefly, before transfection, the cell media were replaced with Opti-MEM (Gibco, Thermo Fisher Scientific, 31985062). DNA was mixed with P3000 Reagent in Opti-MEM and Lipofectamine 3000 Reagent was diluted in Opti-MEM. The DNA mix was added to the Lipofectamine mix and incubated at room temperature for 20 minutes. The DNA/Lipofectamine complex was added to the cells, and media were changed after 12 hours. After 24 hours, as control cells showed high GFP expression, total protein was extracted. Transfected cells were lysed with an IP Lysis buffer (Pierce, Thermo Fisher Scientific, 8788) and protease inhibitors (Roche, MilliporeSigma, 11836170001) and then centrifuged at 13,800*g* for 20 minutes at 4°C. Lysates were stored at -80°C before use for immunoprecipitation or Western blot.

### Immunoprecipitation.

All the steps required for immunoprecipitation were carried out using the DynaMag-Spin Magnet (Invitrogen, Thermo Fisher Scientific, 12320D) following the c-Myc-Tag Magnetic IP/Co-IP Kit manufacturer’s instructions (Pierce, Thermo Fisher Scientific, 88844). Briefly, 1 mL anti-Myc antibody–conjugated magnetic beads was incubated overnight at 4°C. After several washes, the adsorbed protein was eluted with 30 μL elution buffer, incubated at 100°C, and run on a SDS/PAGE followed by Western blot analysis with an anti-Myc antibody (Invitrogen, Thermo Fisher Scientific, PA1-981, 1:2000) and anti-HA antibody (Progen, Thermo Fisher Scientific, 12CA5, 1:2000). Membranes were washed and stained with appropriate IR-conjugated secondary antibodies (anti-mouse antibody, IRDye 800CW Goat anti-mouse IgG secondary [926-32210] and anti-rabbit antibody, IRDye 680CW Goat anti-rabbit secondary antibody [926-68071]) and subsequently imaged using the Li-COR Odyssey imaging system.

### Western blot assays.

Western blot assays were performed as per ref. 45. Primary antibodies used are as follows: anti-VDAC (Abcam, ab154856, 1:1000) and anti-BiP (MilliporeSigma, G9043, 1:3,000). HRP-conjugated secondary antibodies were used (Southern Biotech, 4010-04, 1:10,000). Immunoblots were developed using ECL prime (GE Healthcare, GERPN2232) and imaged using a chemiluminescence detector (Vilber Lourmat). Once imaged, the membrane was stripped by incubating in 1X stripping buffer (200 mM glycine, 0.1% SDS, 1% Tween 20 [MilliporeSigma Aldrich, P9416], pH 2.2) twice for 10 minutes, washed in PBST, and stained with Direct blue (MilliporeSigma Aldrich, 212407) as described previously (46) to detect total protein. The blot images were quantified using Image Lab software (Bio-Rad).

### Mitochondrial function.

Oxygen consumption rate was measured in zebrafish embryos using the Seahorse Bioscience XF 24 extracellular flux analyzer, as previously described with slight modifications (47). In brief, 3 dpf *atrogin-1^+/+^* or *atrogin-1^–/–^* larvae were placed into individual wells of a 24-well XF 24 islet plate (catalog 101122-100) in 630 μL E3 media, in a randomized order. An islet capture screen was added to each well to ensure zebrafish embryos remains in the measurement chamber throughout the assay. A calibrated Seahorse XFe24 Extracellular Flux Assay Kit (catalog 102340-100) was used to measure basal respiration, maximal respiration, spare respiratory capacity, and nonmitochondrial oxygen consumption by titrating FCCP, rotenone, and antimycin A (final concentration of 2 μM for each drug following injection). Each parameter was assessed at least 5 times, and the assay was performed in triplicate. Notably, any larvae that had less than 5% changes in oxygen consumption rates following FCCP and/or rotenone and antimycin A injection were excluded from subsequent analyses.

### Untargeted proteomics using nanoLC ESI DIA-MS data and Spectronaut.

Samples were lysed in SDS lysis buffer (5% w/v sodium dodecyl sulphate, 100 mM HEPES, pH 8.1), heated at 95°C for 10 minutes and then probe-sonicated before measuring the protein concentration using the BCA method. The lysed samples were denatured and alkylated by adding TCEP (Tris(2-carboxyethyl) phosphine hydrochloride) and CAA (2-Chloroacetamide) to a final concentration of 10 mM and 40 mM, respectively, and the mixture was incubated at 55°C for 15 minutes. Sequencing grade trypsin was added at an enzyme-to-protein ratio of 1:50 and incubated overnight at 37°C after the proteins were trapped using S-Trap mini columns (Profiti). Tryptic peptides were sequentially eluted from the columns using (a) 50 mM TEAB, (b) 0.2% formic acid, and (c) 50% acetonitrile, 0.2% formic acid. The fractions were pooled and concentrated in a vacuum concentrator prior to MS analysis.

Using a Dionex UltiMate 3000 RSLCnano system equipped with a Dionex UltiMate 3000 RS autosampler, an Acclaim PepMap RSLC analytical column (75 μm × 50 cm, nanoViper, C18, 2 μm, 100 Å; Thermo Fisher Scientific), and an Acclaim PepMap 100 trap column (100 μm × 2 cm, nanoViper, C18, 5 μm, 100Å; Thermo Fisher Scientific), the tryptic peptides were separated by increasing concentrations of 80% acetonitrile/0.1% formic acid at a flow of 250 nL/min for 158 minutes and analyzed with a QExactive HF mass spectrometer (Thermo Fisher Scientific operated in data-independent acquisition [DIA] mode; 43 sequential DIA windows [isolation width, 14 *m/z*] were acquired [375–975 *m/z*; resolution, 15.000; AGC target, 2 × 10^5^; maximum IT, 22 ms; HCD Collision energy, 27%) following a full MS1 scan (resolution, 60.000; AGC target, 3 × 10^6^; maximum IT, 54 ms; scan range, 375–1,575 *m/z*). Acquired DIA data were evaluated in Spectronaut 14 (Biognosys) using an in-house spectral library.

### Birefringence assays.

Skeletal muscle birefringence was examined using an Abrio polarizing microscope as per previously established protocols (48)

### Movement assays.

The Zebrabox was used to determine the distance and time swum by 6 dpf larvae as per ref. 25. Average speed was calculated by dividing total distance by time.

### Drug concentrations.

All drug treatments were done on 3 dpf larvae for 3 days, with the drug changed in each day. Rotenone used to inhibit mitochondrial complex 1 was used at 10 nM. HM03, a BiP-specific inhibitor was used at 1 μM.

### Statistics.

The GraphPad Prism statistics package was used to analyze data in this study. The number of independent biological replicates (number of lays from different parents) examined for each experiment, the number of fish used within each replicate, the significance tests used, and the associated *t*/*F* value, degrees of freedom, and exact *P* values obtained are detailed in Supplemental Tables 1 and 2. Statistical tests included 1-way ANOVA with Tukey’s multiple correction post hoc test, 2-way ANOVA with Šidák’s multiple correction post hoc test, χ^2^ test, unpaired 2-tailed *t* test, and Fisher’s exact test. *P* values of less than 0.05 were considered significant.

### Study approval.

Fish maintenance was carried out as per the standard operating procedures approved by the Monash Animal Ethics Committee under breeding colony licenses ERM14481 and ERM22161. The generation of transgenic and mutant strains was approved by Monash Animal Ethics Committee (approval nos. ERM16963 and ERM22161).

### Data availability.

The mass spectrometry proteomics data have been deposited in the ProteomeXchange Consortium via the PRIDE (49) partner repository, with the data set identifier PXD038406. All other raw data and files for statistical analysis are available in the Supporting Data Values file and at Figshar (https://figshare.com/s/2885f8fd3167271929c8).

## Author contributions

The project was conceptualized by AAR and PDC. Methodology was provided by AAR and MM. Formal analyses were performed by AAR and CH. Investigation was performed by AAR, MM, CH, JM, SFLW, JDH, CS, LBM, AS, RBS, and TEH. The original draft of the manuscript was written by AAR. Reviewing and editing of the manuscript was performed by AAR and PDC. Visualization was provided by AAR. Supervision was performed by AAR, GSL, RBS, and PDC. Project administration was provided by AAR. Funding was acquired by AAR and PDC.

## Supplementary Material

Supplemental data

Supporting data values

## Figures and Tables

**Table 1:**
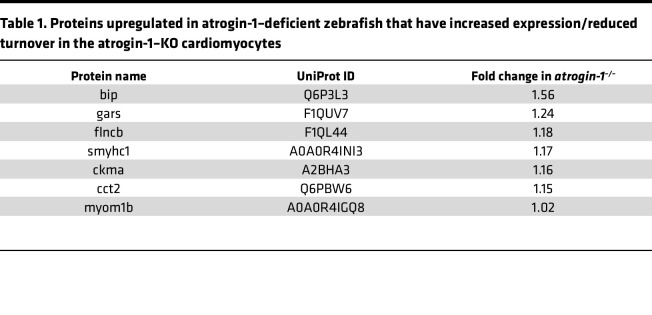
Proteins upregulated in atrogin-1–deficient zebrafish that have increased expression/reduced turnover in the atrogin-1–KO cardiomyocytes

## Version 1 03/26/2024

In-Press Preview

## Version 2 04/22/2024

Electronic publication
