# Supplementary material for: Atrogin-1 promotes muscle homeostasis by regulating levels of endoplasmic reticulum chaperone BiP
Source: JCI Insight. 2024 Mar 26;9(8):e167578. doi: 10.1172/jci.insight.167578 (PMC11141880; doi:10.1172/jci.insight.167578)
Supplement: Supplemental data [file jciinsight-9-167578-s210.pdf]

Atrogin-1 promotes muscle homeostasis by regulating levels of endoplasmic reticulum chaperone BiP.

Avnika A. Ruparelia[1,2,3*], Margo Montandon[1], Jo Merriner[1], Cheng Huang[4], Siew Fen Lisa Wong [1], Carmen Sonntag[1], Justin P. Hardee[3], Gordon S. Lynch[3], Lee B. Miles[1], Ashley Siegel[1], Thomas E. Hall[5], Ralf B. Schittenhelm[4] and Peter D. Currie[1,6*]

[1] Australian Regenerative Medicine Institute, Monash University, Wellington Road, Clayton, VIC 3800, Australia.

[2] Department of Anatomy and Physiology, School of Biomedical Sciences, Faculty of Medicine Dentistry and Health Sciences, University of Melbourne, Melbourne, Victoria, 3010, Australia.

[3] Centre for Muscle Research, Department of Anatomy and Physiology, University of Melbourne, Melbourne, Victoria, 3010, Australia

[4] Monash Proteomics and Metabolomics Facility, Monash Biomedicine Discovery Institute, Monash University, Clayton, Victoria 3800, Australia.

[5] Institute for Molecular Bioscience, University of Queensland, Brisbane, QLD 4072 Australia.

[6] EMBL Australia, Victorian Node, Level 1, 15 Innovation Walk, Monash University, Wellington Road, Clayton, VIC 3800, Australia.

*Co-corresponding authors

Contact for correspondence:

Dr Avnika Ruparelia

Centre for Muscle Research

Level 5W, Medical Building 181

University of Melbourne, Parkville VIC 3010, Australia

Tel: +61 3 8344 8786

avnika.ruparelia@unimelb.edu.au

Prof Peter D. Currie
Australian Regenerative Medicine Institute.
Level 1, 15 Innovation Walk
Monash University, Wellington Road
Clayton VIC 3800.
Australia.
Tel +61 3 99029602
peter.currie@monash.edu

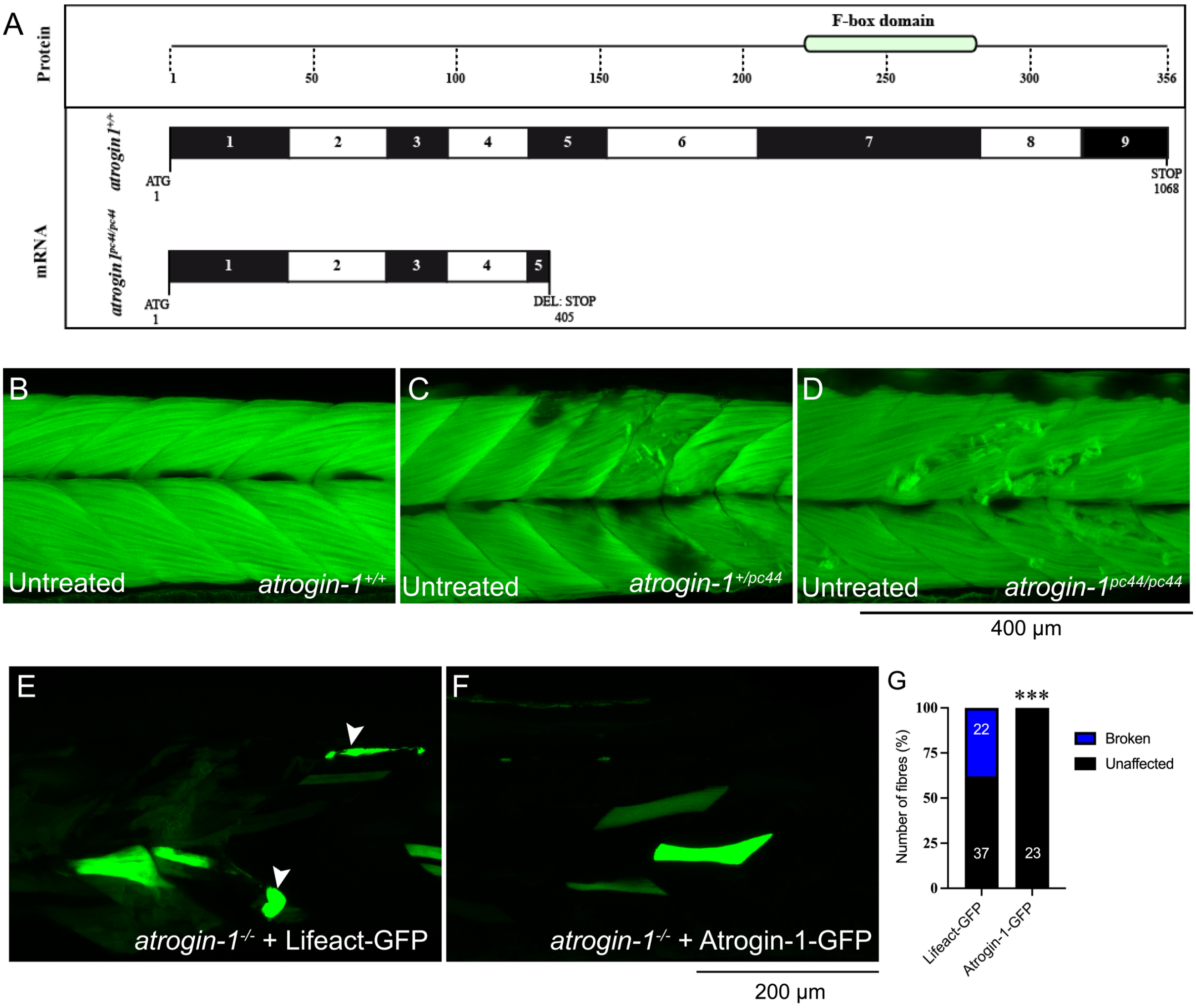

Supplementary Figure 1: Atrogin-1 deficiency results in muscle fibre detachment. (**A**) Schematic of wildtype *atrogin-1* (*atrogin-1*$^{+/+}$) and mutant *atrogin-1* (*atrogin-1*$^{pc44/44}$) protein structure and mRNA sequence, with the mutant predicted to incorporate a premature stop in exon 5. The mutant was generated using Zinc Finger Nuclease technology, resulting in a 7 base pair deletion. (**B-D**) While muscle fibres span the entire length of the somite in *atrogin-1*$^{+/+}$ wildtype larvae, *atrogin-1*$^{+/pc44}$ and *atrogin-1*$^{pc44/pc44}$ mutant larvae display muscle fibre detachment as seen by F-Actin labeling. (**E-F**) While Lifeact-GFP expressing cells in the *atrogin-1*$^{-/-}$ mutant undergo disintegration, expression of Atrogin-1-GFP is sufficient to prevent fibre disintegration. (**G**) Quantification of number of intact and disintegrated cells following Lifeact-GFP or Atrogin-1-GFP expression - as determined using Fisher's exact test. ***$p<0.001$. All experiments performed in triplicates with the total number of fish examined in each replicate is documented in Supplementary Table 2.

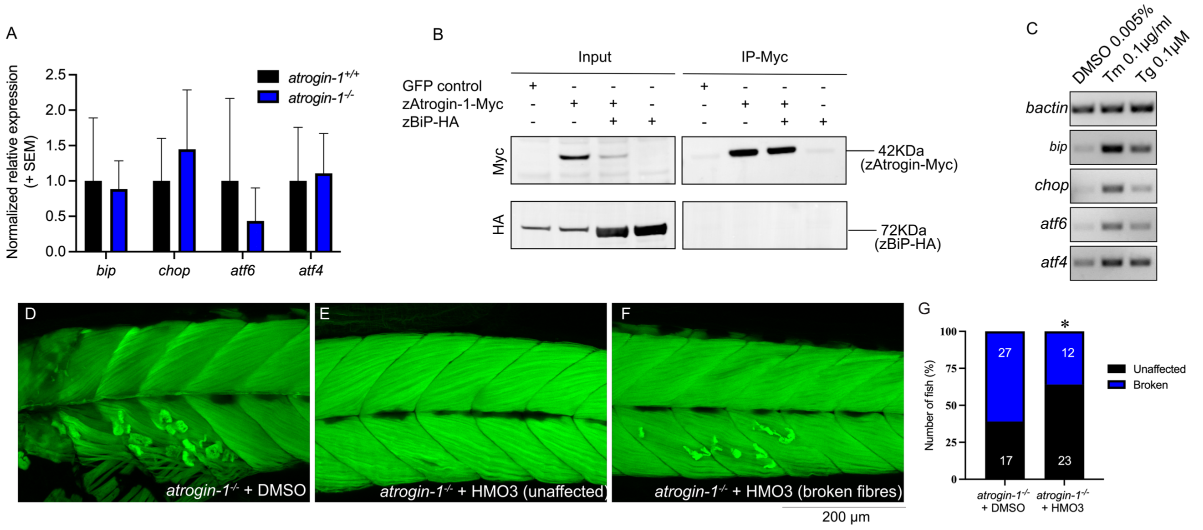

Supplementary Figure 2: Abundance of ER stress markers. (**A**) qRT-PCR analysis showing no significant change in the expression of ER stress genes *bip*, *chop*, *atf6* and *atf4* comparing *atrogin-1*$^{+/+}$ wildtype larvae and *atrogin-1*$^{-/-}$ mutants – as determined using a one way ANOVA with Tukey's multiple correction post hoc test. Error bars represent +/- SEM. (**B**) Western blot for Myc and HA levels in whole cell lysates (input) and lysates following Myc immunoprecipitation (IP-Myc). While Myc-atrogin-1 was enriched in the Myc-atrogin-1 and Myc-atrogin-1 and BiP-HA transfected cells, indicating successful pulldown, no HA-tagged BiP was detected in any of the immunoprecipitated lysates (**C**) RT-PCR reveals that ER stress inducers Tunicamycin (Tm) or Thapsigargin (Tg) induced the expression of UPR genes *bip*, *chop*, *atf6* and *atf4. (*~~**D-F**~~) Muscle phenotypes in 6 dpf DMSO or HM03 treated *atrogin-1*$^{-/-}$ mutants following incubation in methyl cellulose. (**G**) HM03 treatment resulted in a reduction in the number of fish displaying fibre disintegration as determined using Fisher's exact test. *$p < 0.05$. All experiments performed in triplicates with the total number of fish examined in each replicate is documented in Supplementary Table 2.

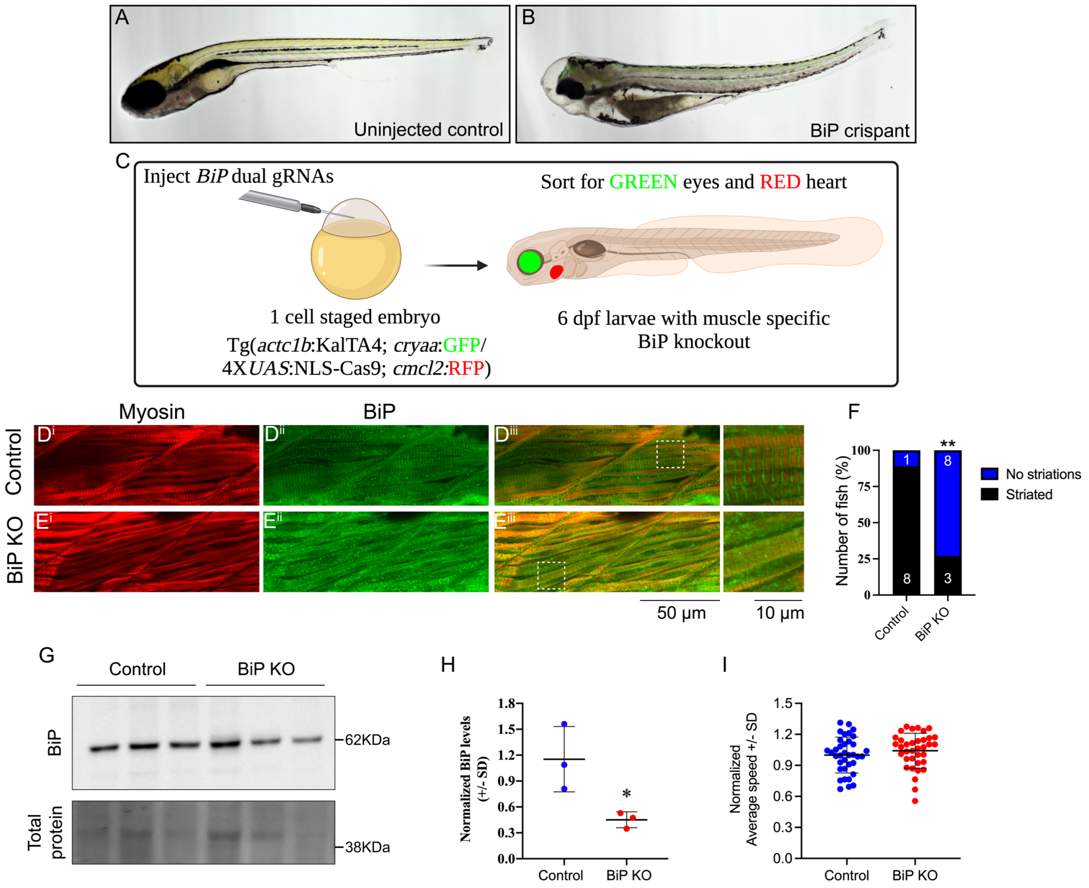

Supplementary Figure 3. **Generation and validation of BiP deficient models.** (**A-B**) Representative bright field image of a 6 dpf BiP crispant larvae display striking phenotypes including odema in the brain and heart with no adverse phenotypes seen in the uninjected control. (**C**) Schematic of muscle specific knockout strategy used. (**D-E**) Single slice confocal images showing striated ER-like BiP localization in control KalTA4 larvae displayed which is lost in BiP KO larvae following muscle specific mutagenesis of BiP. (**F**) Quantification of the proportion of control and BiP KO fish altered BiP protein localization. BiP KO fish show a significant increase in the number of fish displaying a loss in striations – as determined using Fisher's exact test. (**G**) Representative western blot images for BiP, and total protein direct blue stain, on whole cell protein lysates obtained from three independent biological replicates, each containing multiple control KalTA4 negative larvae or BiP KO larvae. (**H**) Quantification of BiP levels normalized to total protein with BiP KO larvae displaying a significant reduction compared to control – as determined using an unpaired t test. Error bars represent +/- SD. (I) Normalized average speed of 6 dpf control or BiP KO larvae - analysed using a one-way ANOVA with Sidak's multiple correction post hoc test. Error bars represent +/- SD. **p < 0.01. All experiments performed in triplicates with the total number of fish examined in each replicate is documented in Supplementary Table 2.

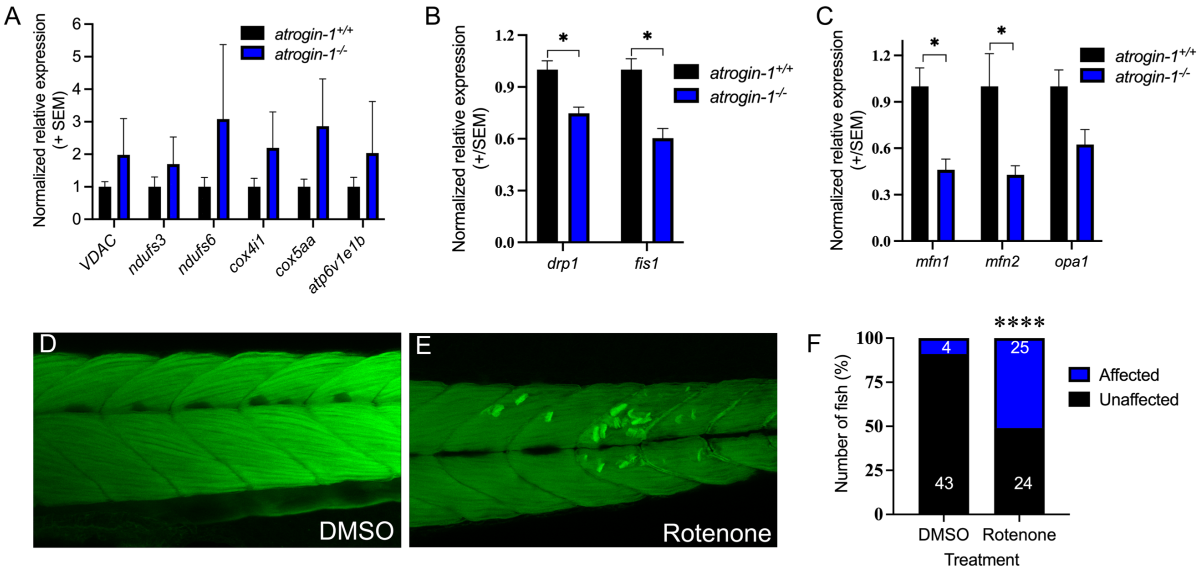

Supplementary Figure 4: Mitochondrial inhibition results in muscle fibre detachment. (**A**) qRT-PCR analysis showing no significant change in the expression of mitochondrial genes *VDAC*, *ndufs3*, *ndufs6*, *cox4i1*, *cox5aa* and *atp6v1e1b* comparing *atrogin-1*$^{+/+}$ wildtype larvae and *atrogin-1*$^{-/-}$ mutants - as determined using a one way ANOVA with Tukey's multiple correction post hoc test. Error bars represent ±SEM for three replicate experiments with each experiment comprising a pooled sample of at least 5 fish. No significant changes identified as determined using a two way ANOVA with Sidak's multiple correction post hoc test. (**B**) qRT-PCR analysis reveals a significant downregulation of mitochondrial fission genes *drp1* and *fis1* in *atrogin-1*$^{-/-}$ mutants, as determined using an unpaired t test. Error bars represent ±SEM for three replicate experiments with each experiment comprising a pooled sample of at least 5 fish. (**C**) qRT-PCR analysis reveals a significant downregulation of mitochondrial fusion genes *mfn1, mfn2 and opa1* in *atrogin-1*$^{-/-}$ mutants, as determined using an unpaired t test. Error bars represent ±SEM for three replicate experiments with each experiment comprising a pooled sample of at least 5 fish. (**D-E**) Inhibition of complex 1 with Rotenone results in muscle fibre detachment following methyl cellulose incubation, which is not apparent in DMSO treated larvae – as seen using an F-Actin stain. (**F**) Graph showing the percentage of affected DMSO and Rotenone treated larvae with the latter having a significant increase in the proportion of fish displaying the muscle fibre detachment as determined using a Fisher's exact test. *$p<0.05$, ****$p < 0.0001$. All experiments performed in triplicates with the total number of fish examined in each replicate is documented in Supplementary Table 2.

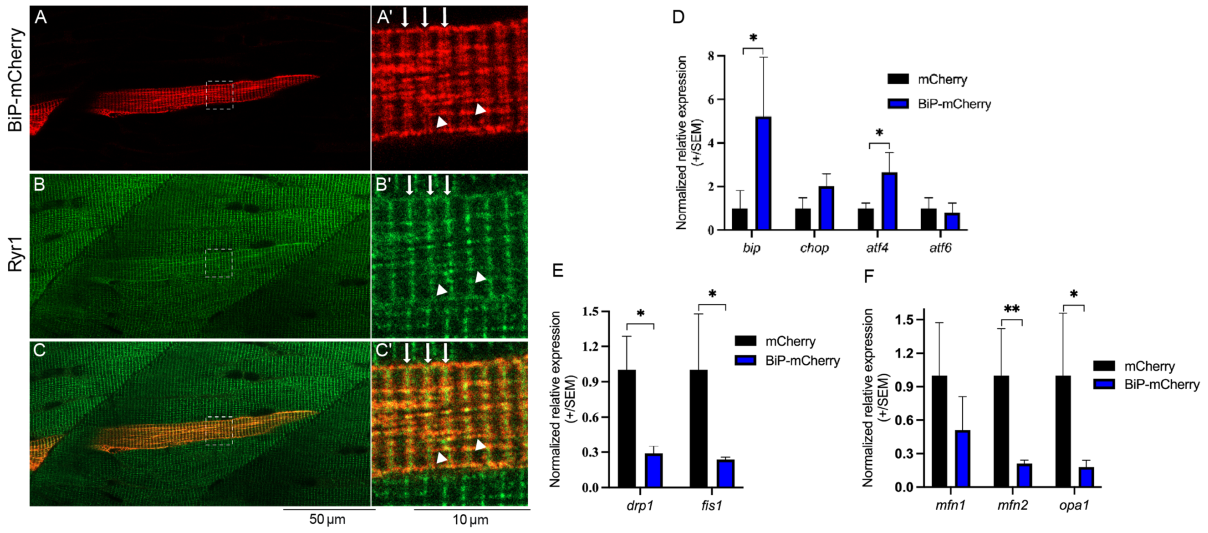

Supplementary Figure 5. Overexpression of BiP results in altered mitochondrial function (**A-C**) BiP-mcherry localizes to the terminal cristae of the SR (arrows) - a structure directly adjacent to the Ryr1 labelled T-tubules, and more generally within the SR network (arrowheads). (**D**) qRT-PCR analysis showing a significant increase in levels of ER stress genes *bip* and *atf4* in 2 dpf larvae injected with BiP-mCherry RNA compared to mCherry RNA injected fish - as determined using an unpaired t test. Error bars represent ±SEM for three replicate experiments with each experiment comprising a pooled sample of at least 5 fish (**E**) qRT-PCR analysis reveals a significant downregulation of mitochondrial fission genes *drp1* and *fis1* in BiP-mCherry RNA injected larvae, as determined using an unpaired t test. Error bars represent ±SEM for three replicate experiments with each experiment comprising a pooled sample of at least 5 fish. (**F**) qRT-PCR analysis reveals a significant downregulation of mitochondrial fusion genes *mfn1, mfn2 and opa1* in BiP-mCherry RNA injected larvae, as determined using an unpaired t test. Error bars represent ±SEM for three replicate experiments with each experiment comprising a pooled sample of at least 5 fish. *$p<0.05$, **$p < 0.01$. All experiments performed in triplicates with the total number of fish examined in each replicate is documented in Supplementary Table 2.

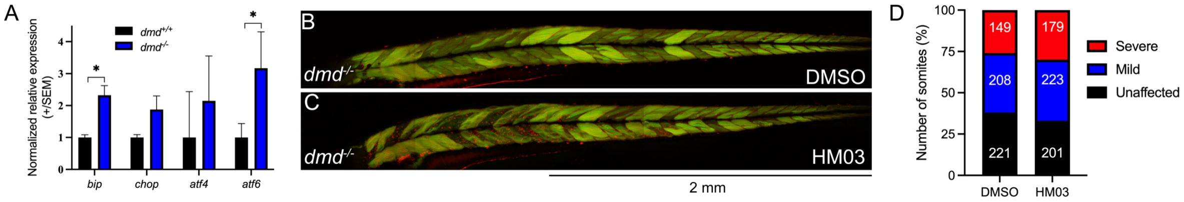

Supplementary Figure 6. BiP inhibition has no effect on muscle integrity. (**A**) qRT-PCR analysis showing a significant increase in levels of ER stress genes *bip* and *atf6* in 6 dpf *dmd*$^{-/-}$ mutants, as determined using an unpaired t test. Error bars represent ±SEM for three replicate experiments with each experiment comprising a pooled sample of at least 5 fish (**B-C**) Live images of 6 dpf DMSO or HM03 treated *dmd*$^{-/-}$ on the (Tg(*actc1b*:Lifeact-GFP);Tg(*actc1b*:CAAX-mCherry) background, whereby the actin filaments within the muscle fibres are labelled with GFP and membrane and t-tubules with mCherry. DMSO treated and HM03 treated mutants display similar severities in muscle fibre detachment. (**D**) Quantification of the number of fish displaying no muscle fibre detachment, mild or severe detachment phenotypes with no significant difference between DMSO and HM03 observed – as per chi squared test. All experiments performed in triplicates with the total number of fish examined in each replicate is documented in Supplementary Table 2.

Supplementary Table 1: Details of statistical tests used for each Figure.

Figure	Description	Test used	t/F value	degrees of freedom (df)	Multiple comparison	p values (adjusted)
1B	qPCR: atrogin-1	One way ANOVA with Tukey's multiple correction post hoc test	-	6	atrogin-1+/+ vs atrogin-1+/-	0.811
					atrogin-1+/+ vs atrogin-1-/-	0.04
1D	3 dpf untreated	Chi squared test	0.7482	2	N/A	0.6879
1F	3 dpf methyl	Chi squared test	15.58	2	N/A	0.0004
1H	6 dpf untreated	Chi squared test	6.867	2	N/A	0.0323
1J	6 dpf methyl	Chi squared test	21.45	2	N/A	<0.0001
3C	Western blot: BiP	Unpaired t test	2.171	10	N/A	0.0276
3G	Fibre integrity	Chi squared test	8.997	2	N/A	0.0111
3J	Muscle rescue	Fisher's exact test	N/A	N/A	N/A	0.0448
4A	KEGGS enrichment analyses	modEnrichr	N/A	N/A	Oxidative phosphorylation	2.05
					Oocyte meiosis	1.69
					Cell cycle	1.69
					Focal adhesion	1.32
					Ribosome	1.23
					Regulation of actin cytoskeleton	1.23
					Salmonella infection	1.22
					Tight junction	1.22
					Aminoacyl-tRNA biosynthesis	0.99
					Spliceosome	0.82
					Tyrosine metabolism	0.79
					Glycerolipid metabolism	0.37

					Arginine and proline metabolism	0.37
					PPAR signaling pathway	0.37
					Lysosome	0.37
4D	Western blot: VDAC	Unpaired t test	2.484	10	atrogin-1+/+ vs atrogin-1-/-	0.0162
4G	Mitochondrial dynamics	Fisher's exact test	N/A	N/A	N/A	<0.0001
4J	Basal respiration	Unpaired t test	2.232	40	atrogin-1+/+ vs atrogin-1-/-	0.0313
4K	Maximum respiation	Unpaired t test	2.583	40	atrogin-1+/+ vs atrogin-1-/-	0.0136
5D	Mitochondrial dynamics	Chi squared test	10.92	2	N/A	0.0042
5G	BiP-mCherry - mitochondrial dynamics	Fisher's exact test	N/A	N/A	N/A	<0.0001
5J	Mitochondrial dynamics rescue	Fisher's exact test	N/A	N/A	N/A	0.0002
6B	Western blot: BiP	Two way ANOVA with Sidak's multiple correction post hoc test	0.3787	8	2 dpf - dmd+/+ vs dmd-/-	0.9186
			3.782		4 dpf - dmd+/+ vs dmd-/-	0.0107
6G	dmd birefringence	One way ANOVA with Tukey's multiple correction post hoc test	7.945	46	dmd+/+; atrogin-1+/+ vs. dmd-/-; atrogin-1+/+	<0.0001
			0.8675		dmd+/+; atrogin-1+/+ vs. dmd+/+; atrogin-1-/-	0.9273
			11.05		dmd+/+; atrogin-1+/+ vs. dmd-/-; atrogin-1-/-	<0.0001
			9.671		dmd-/-; atrogin-1+/+ vs. dmd+/+; atrogin-1-/-	<0.0001
			3.964		dmd-/-; atrogin-1+/+ vs. dmd-/-; atrogin-1-/-	0.0359

			12.92		dmd+/+; atrogin-1-/- vs. dmd-/-; atrogin-1-/-	<0.0001
			6.329		dmd+/+; atrogin-1+/+ vs. dmd-/-; atrogin-1+/+	0.0002
			3.285		dmd+/+; atrogin-1+/+ vs. dmd+/+; atrogin-1-/-	0.1022
			10.1		dmd+/+; atrogin-1+/+ vs. dmd-/-; atrogin-1-/-	<0.0001
			3.128		dmd-/-; atrogin-1+/+ vs. dmd+/+; atrogin-1-/-	0.1297
			3.948		dmd-/-; atrogin-1+/+ vs. dmd-/-; atrogin-1-/-	0.0331
6H	dmd zebrabox	One way ANOVA with Tukey's multiple correction post hoc test	7.036	73	dmd+/+; atrogin-1-/- vs. dmd-/-; atrogin-1-/-	<0.0001
			11.62		GFP:dmd+/+ vs. GFP:dmd-/-	<0.0001
			1.011		GFP:dmd+/+ vs. Atrogin-1-IRES-GFP:dmd+/+	0.8967
			8.517		GFP:dmd+/+ vs. Atrogin-1-IRES-GFP:dmd-/-	<0.0001
			11.65		GFP:dmd-/- vs. Atrogin-1-IRES-GFP:dmd+/+	<0.0001
6O	Overexpression birefringence	Two way ANOVA with Sidak's multiple correction post hoc test	3.526	87	GFP:dmd-/- vs. Atrogin-1-IRES-GFP:dmd-/-	0.004

			8.802		Atrogin-1-IRES-GFP:dmd+/+ vs. Atrogin-1-IRES-GFP:dmd-/-	<0.0001
7D	HM03 birefringence	Two way ANOVA with Sidak's multiple correction post hoc test	2.693	81	dmd+/+; dmd-/- DMSO	0.0171
			4.759		dmd+/+; dmd-/- HM03	<0.0001
7E	HM03 zebrabox	Two way ANOVA with Sidak's multiple correction post hoc test	6.31	114	DMSO:dmd+/+ vs. DMSO:dmd-/-	0.0001
			2.812		DMSO:dmd+/+ vs. HM03:dmd+/+	0.1985
			1.477		DMSO:dmd+/+ vs. HM03:dmd-/-	0.7239
			3.278		DMSO:dmd-/- vs. HM03:dmd+/+	0.0999
			4.985		DMSO:dmd-/- vs. HM03:dmd-/-	0.0034
			1.443		HM03:dmd+/+ vs. HM03:dmd-/-	0.7377
Supp 1A	atrogin-1 overexpression rescue	Fisher's exact test	N/A	N/A	N/A	0.0062
Supp 2A	qPCR: ER stress	One way ANOVA with Tukey's multiple correction post hoc test	0.1517	16	bip	0.9998
			0.4586		chop	0.9854
			1.035		atf6	0.7814
			0.1274		atf4	>0.9999
Supp 2G	atrogin-1 + HM03	Fisher's exact test	N/A	N/A	N/A	0.0212
Supp 3F	BiP localization	Fisher's exact test	N/A	N/A	N/A	0.0098
Supp 3G-H	Western blot: BiP	Unpaired t test	3.119	4	Control vs BiP KO	0.0356

Supp 3I	BiP KO zebrabox	Unpaired t test	1.045	70	Control vs BiP KO	0.2996
Supp 4A	qPCR: OXPHOS	Two way ANOVA with Sidak's multiple correction post hoc test	1.302	24	VDAC	0.7482
			1.006		ndufs3	0.9048
			2.143		ndufs6	0.2294
			1.5		cox4i1	0.614
			2.003		cox5aa	0.2953
			1.355		atp6v1e1b	0.7134
Supp 4B	qPCR: mito fission (atrogin-1)	Unpaired t test	N/A	N/A	drp1	0.02823719
			N/A	N/A	fis1	0.01793509
Supp 4C	qPCR: mito fussion (atrogin-1)	Unpaired t test	N/A	N/A	mfn1	0.02472485
			N/A	N/A	mfn2	0.04296338
			N/A	N/A	opa1	0.07585907
Supp 4F	Rotenone	Fisher's exact test	N/A	N/A	N/A	
Supp 5D	qPCR: ER stress (BiP-mCherry)	One way ANOVA with Tukey's multiple correction post hoc test	0.1517	16	bip	0.04264868
			0.4586		chop	0.10254815
			1.035		atf6	0.35412295
			0.1274		atf4	0.02683237
Supp 5E	qPCR: mito fission (BiP-mCherry)	Unpaired t test	N/A	N/A	drp1	0.03596549
			N/A	N/A	fis1	0.03836627
Supp 5F	qPCR: mito fussion (BiP-mCherry)	Unpaired t test	N/A	N/A	mfn1	0.25627879
			N/A	N/A	mfn2	0.00974165
			N/A	N/A	opa1	0.01080308
Supp 6A	qPCR: ER stress (dmd)	Unpaired t test	3.806	10	bip	0.010326
			2.091		chop	0.122125
			1.594		atf4	0.141933
			4.533		atf6	0.004341
Supp 6D	DMD drugs	Chi squared test	3.686	2	DMSO vs HM03	0.1583

Supplementary Table 2: Sample numbers for each Figure.

Figure	Description	Number of independent replicates	Group	Total fish/fibre numbers
1B	qPCR: atrogin-1	3	atrogin-1+/+	>5 larvae/replicate
			atrogin-1+/pc43	>5 larvae/replicate
			atrogin-1pc43/pc43	>5 larvae/replicate
1C-D	3 dpf untreated	3	atrogin-1+/+	16
			atrogin-1+/pc43	38
			atrogin-1pc43/pc43	12
1E-F	3 dpf methyl	3	atrogin-1+/+	17
			atrogin-1+/pc43	52
			atrogin-1pc43/pc43	23
1G-H	6 dpf untreated	3	atrogin-1+/+	27
			atrogin-1+/pc43	37
			atrogin-1pc43/pc43	12
1I-J	6 dpf methyl	3	atrogin-1+/+	24
			atrogin-1+/pc43	50
			atrogin-1pc43/pc43	25
3A	Proteomics	3	atrogin-1+/+	8-12 larvae/replicate
			atrogin-1pc43/pc43	8-12 larvae/replicate
3B-C	Western blot: BiP	6	atrogin-1+/+	>5 larvae/replicate
			atrogin-1pc43/pc43	>5 larvae/replicate
3D-G	Fibre detachment	3	DMSO	19
			Tm	17
			Tg	19
3H-J	Muscle fibre rescue	3	Control	18
			BiP KO	19
4C-D	Western blot: VDAC	6	atrogin-1+/+	>5 larvae/replicate
			atrogin-1pc43/pc43	>5 larvae/replicate
4E-G	Mitochondrial dynamics	3	atrogin-1+/+	57 fibres
			atrogin-1pc43/pc43	51 fibres

4H-I	Electron miscroscopy	3	atrogin-1+/+	4 fish
			atrogin-1pc43/pc43	4 fish
4J-K	Mitochondrial function	3	atrogin-1+/+	18 fish
			atrogin-1pc43/pc43	24 fish
5A-C	Mitochondrial dynamics	3	DMSO	40 fibres
			Tm	46 fibres
			Tg	37 fibres
5E-G	BiP-mCherry - mitochondrial dynamics	3	mCherry	50 fibres
			BiP-mCherry	76 fibres
5H-J	Mitochondrial dynamics rescue	3	Control	89 fibres
			BiP KO	49 fibres
6A-B	Western blot: BiP	3	dmd+/+	>5 larvae/replicate
			dmdpc2/pc2	>5 larvae/replicate
6C-G	dmd birefringence	3	dmd+/+; atrogin-1+/+	9
			dmdpc2/pc2; atrogin-1+/+	16
			dmd+/+; atrogin-1pc43/pc43	12
			dmdpc2/pc2; atrogin-1pc43/pc43	13
6H	dmd zebrabox	6	dmd+/+; atrogin-1+/+	18
			dmdpc2/pc2; atrogin-1+/+	20
			dmd+/+; atrogin-1pc43/pc43	20
			dmdpc2/pc2; atrogin-1pc43/pc43	19
6K-O	Overexpression birefringence	3	dmd+/+ + GFP	22
			dmd+/+ + IRES GFP	16
			dmdpc2/pc2 + GFP	25
			dmdpc2/pc2 + IRES GFP	28
7A-D	HM03 birefringence	4	dmd+/+ + DMSO	19
			dmd+/+ + HM03	18

			dmdpc2/pc2 + DMSO	23
			dmdpc2/pc2 + HM03	25
7E	HM03 zebrabox	3	dmd+/+ + DMSO	27
			dmd+/+ + HM03	26
			dmdpc2/pc2 + DMSO	34
			dmdpc2/pc2 + HM03	31
Supp 1B-D	atrogin-1pc44/pc44	1	atrogin-1+/+	6
			atrogin-1+/pc44	16
			atrogin-1pc44/pc44	7
Supp 1E-G	atrogin-1 overexpression rescue	1	Lifeact-GFP	90 lrvae
			atrogin-1-GFP	22 larvae
Supp 2A	qPCR: ER stress	3	atrogin-1+/+	>5 larvae/replicate
			atrogin-1+/pc43	>5 larvae/replicate
Supp 2D-G	atrogin-1 + HM03	3	atrogin-1-/- + DMSO	44 larbae
			atrogin-1-/- + HM03	36 larvae
Supp 3F	BiP localization	1	control	9
			BiP KO	11
Supp 3G-H	Western blot: BiP	3	control	>5 larvae/replicate
			BiP KO	>5 larvae/replicate
Supp 3I	BiP KO zebrabox	3	control	36 larvae
			BiP KO	36 larvae
Supp 4A-C	qPCR: OXPHOS, mito fission and fusion	3	atrogin-1+/+	>5 larvae/replicate
			atrogin-1pc43/pc43	>5 larvae/replicate
Supp 4D-F	Rotenone	3	DMSO	46
			Rotenone	49
Supp 5D-F	qPCR: OXPHOS, mito fission and fusion	3	mCherry	>5 larvae/replicate
			BiP-mCherry	>5 larvae/replicate
Supp 6A	qPCR: ER stress	6	dmd+/+	>5 larvae/replicate
			dmdpc2/pc2	>5 larvae/replicate
Supp 5A-C	DMD drugs	3	Control	578 fibres
			HM03	603 fibres

Supplementary Table 3: Sequences of primers used

Primer	Sequence
atrogin-1 gRNA 1	ggacaagactggcggtctcc
atrogin-1 targetting stop cassette	CTTggacaagactggcggtcGTCATGGCGTTTAAACCTTAATTAAGCTGTTGTAGtccTGGTCAAAGCTGGGTTA
atrogin-1pc43/pc43 genotyping F	gctgcgcacttttatcatca
atrogin-1pc43/pc43 genotyping F	ATCCTCCGTTGACCAACACT
dmdpc2/pc2 genotyping F	aatgcctgtaaacaaatgtgtctgt
dmdpc2/pc2 genotyping R	ccttgccatgttaacccaaatt
BiP gRNA 1	CCGCATCACTCCGTCATACG
BiP gRNA 2	CACAAACGGAGACACTCACC
atrogin-1 qPCR F	ggaaagggttgtgcagaaag
atrogin-1 qPCR R	ctgctgccactgcagtatgt
b-actin_F	GCATTGCTGACCGTATGCAG
b-actin_R	GATCCACATCTGCTGGAAGGTGG
GAPDH_F	AGCACTGTTCATGCCATCAC
GAPDH_R	GCTCAGGAATTACTTTGCCTACA
VDAC1_F	CATTGCAGCCAAATACCAAA
VDAC1_R	TCAAACTCCAGACCCAAACC
ndufs3_F	GCCTCCTTGGTCAGATTTGT
ndufs3_R	GGGAGAATCTCTGCGACGTA
ndufs6_F	CAGCAGTTCCAGTTCAGCAG
ndufs6_R	CCAGGTTGATTGCAAAGTTCT
cox4i1_F	TACGGCATTTCGTCTTGTTG
cox4i1_R	CCCAGGATCCCTTCTCTTTC
cox5aa_F	ACGGATGAGGAGTTTGATGC
cox5aa_R	TGGCCAGATCGTCTAACCTC
atp6v1e1b_F	CAATGAAAAAGCCGAGGAGA
atp6v1e1b_R	GTCATCTCTGGCCTTCAGGA
bip_qRT_F	aagaggccgaagagaaggac

bip_qRT_R	agcagcagagcctcgaaata
atf4_qRT_F	ttagcgattgctccgatagc
atf4_qRT_R	gctgcggttttattctgctc
atf6_qRT_F	ctgtggtgaaacctccacct
atf6_qRT_R	catggtgaccacaggagatg
chop_qRT_F	aaggaaagtgcaggagctga
chop_qRT_R	tcacgctctccacaagaaga
BiP gRNA 1	CCGCATCACTCCGTCATACG
BiP gRNA 2	CACAAACGGAGACACTCACC
drp1_F	AGCCAGTCAGGTGATCGCCGA
drp1_R	CGCAGGGTTCGCGTGAAGGG
fis1_F	AGATGGTTTAGTCGGCATGG
fis1_R	TCAGGCCTCCTTGTGTTTTT
mfn1_F	GACCGCATCTTCTTCGTCTC
mfn1_R	TGTGCTGCTCAAACTTGGTC
mfn2_F	AAAGCCAAACTGCTCAGGAA
mfn2_R	GGCGGAAAGAACAACGAATA
opa1_F	AGACTGGAAGCAGAGGTGGA
opa1_R	TTGCGCACTGTAGTGACCTC
mitoF1_pME	GGGGACAAGTTTGTACAAAAAAGCAGGCTGCCACCatgTCTGGACTTCTGAGGGGACT
GFPstopR1_pME	GGGGACCACTTTGTACAAGAAAGCTGGGTGttaCTTGTACAGCTCGTCCATGC
pME_zAtrogin1_F	GGGGACAAGTTTGTACAAAAAAGCAGGCTTCGgccaccATGCCGTTTCTTGGACAAGACTGGC
pME_zAtrogin1_R	GGGGACCACTTTGTACAAGAAAGCTGGGTCCGCTAAAACTTGAAGAGGTTGATGAA
